# Multiple types of navigational information are independently encoded in the population activities of the dentate gyrus neurons

**DOI:** 10.1073/pnas.2106830119

**Published:** 2022-08-05

**Authors:** Tomoyuki Murano, Ryuichi Nakajima, Akito Nakao, Nao Hirata, Satoko Amemori, Akira Murakami, Yukiyasu Kamitani, Jun Yamamoto, Tsuyoshi Miyakawa

**Affiliations:** ^a^Division of Systems Medical Science, Center for Medical Science, Fujita Health University, Toyoake 470-1192, Japan;; ^b^Department of Synthetic Chemistry and Biological Chemistry, Graduate School of Engineering, Kyoto University, Kyoto 615-8510, Japan;; ^c^Institue for the Advanced Study of Human Biology, Kyoto University, Kyoto 606-8501, Japan;; ^d^Graduate School of Informatics, Kyoto University, Kyoto 606-8501, Japan;; ^e^Department of Psychiatry, University of Texas Southwestern Medical Center, Dallas, TX 75390

**Keywords:** dentate gyrus, calcium imaging, machine learning

## Abstract

The dentate gyrus (DG) plays critical roles in cognitive functions, such as learning, memory, and spatial coding, and its dysfunction is implicated in various neuropsychiatric disorders. However, it remains largely unknown how information is represented in this region. Here, we recorded neuronal activity in the DG using Ca^2+^ imaging in freely moving mice and analyzed this activity using machine learning. The activity patterns of populations of DG neurons enabled us to successfully decode position, speed, and motion direction in an open field, as well as current and future location in a T-maze, and each individual neuron was diversely and independently tuned to these multiple information types. Our data also showed that each type of information is unevenly distributed in groups of DG neurons, and different types of information are independently encoded in overlapping, but different, populations of neurons. In alpha-calcium/calmodulin-dependent kinase II (αCaMKII) heterozygous knockout mice, which present deficits in spatial remote and working memory, the decoding accuracy of position in the open field and future location in the T-maze were selectively reduced. These results suggest that multiple types of information are independently distributed in DG neurons.

## Significance

In this study, we found that multiple types of information (position, speed, and motion direction in an open field and current and future location in a T-maze) are independently encoded in the overlapping, but different, populations of dentate gyrus (DG) neurons. This computational nature of the independent distribution of information in neural circuits is newly found not only in the DG, but also in other hippocampal regions.

The dentate gyrus (DG) in the hippocampus has been implicated in cognitive functions such as pattern separation (1, 2), contextual encoding (3, 4), and place recognition (3–7), and its dysfunction is suggested to be associated with various neuropsychiatric disorders, such as epilepsy (8), schizophrenia (9, 10), and Alzheimer’s disease (8). The DG receives excitatory input from layer II neurons in the entorhinal cortex (EC) (11, 12) and sends output to Cornu Ammonis 3 (CA3) pyramidal cells (4, 5). The EC receives multimodal sensory information from other brain regions and contains neurons with distinct functional properties, including grid (13), border (14), speed (15, 16), and head-direction cells (17). Likewise, the hippocampal CA regions receive information from the DG and contain neurons that represent place (18), locomotion speed (16), episodic memory (19), time (20), and novel spatial experience (21, 22). Therefore, the DG is thought to be involved in the processing and integration of a variety of information (23).

Despite its wide-ranging functions, the DG is unusual among hippocampal regions in that only a small population of principal DG neurons are thought to be active in a given environment (24, 25). In addition, several studies have reported that most DG neurons exhibit poor tuning to position (6) and movement speed (26). This paradox has led to a significant interest in how information is represented in the population activity patterns of DG neurons (7).

Here, to unveil important functional characteristics of DG neurons, we performed Ca^2+^ imaging using a microendoscope in freely moving mice, which enables us to record activity from a large population of neurons (27, 28). We recorded neural activity of the dorsal DG (dDG) that is thought to be relatively more involved in the exploratory behavior and contextual memory encoding than in the ventral DG (29, 30). Then, we analyzed how individual DG neurons are involved in encoding position, speed, and motion direction in an open field (OF), as well as current and future location (left or right) in a T-maze. Even if the majority of individual neurons are poorly tuned to these types of information, neurons may encode information by the population activity patterns (7). Therefore, by using machine-learning methods, we also asked if these types of information are encoded in the population activity patterns of DG neurons. We then investigated how these types of information are distributed in the populations of DG neurons. Concurrently, to assess how these neural representations might be altered in a disease model, we carried out the same imaging and analysis in heterozygous alpha-calcium/calmodulin-dependent kinase II knockout (αCaMKII^+/−^) mice. The αCaMKII^+/−^ mice exhibit an array of behavioral abnormalities, including locomotor hyperactivity, impaired working and remote memory, abnormal social/aggressive behavior, and exaggerated infradian rhythms (31–35). We have reported that these mice have an endophenotype called “immature DG (iDG),” in which neurons show properties similar to immature neurons, such as altered expression of maturation-related genes, decreased induction of some of the immediate early genes (*cFos* and *Arc*), increased membrane excitability, and weak synaptic plasticity at mossy-fiber–CA3 synapses (31, 34). By studying how neural representation of information is altered in the DG of these mice, we further aimed to gain insight into how multiple types of information are represented in the population activities of dDG neurons in normal and abnormal conditions.

## Results

### Activity Patterns of dDG Neurons Are Tuned to the Position and Speed of the Mouse.

**Fig. 1.**
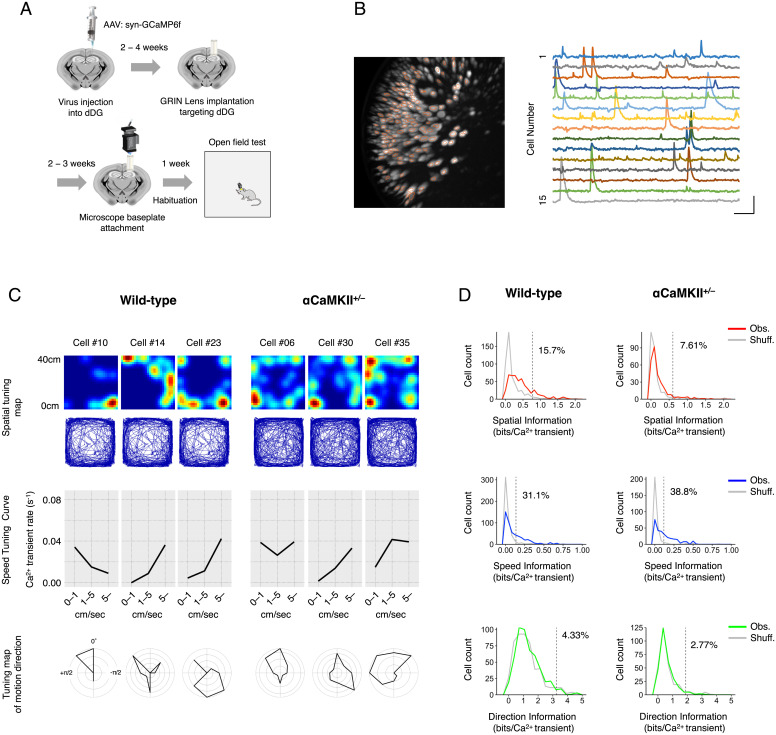
Activity patterns of dDG neurons are slightly tuned to the position and speed of the mouse. (*A*) Schematic of the experimental procedures. GCaMP6f was expressed in the dDG of mice by an AAV injection. At least 2 wks after viral injection, a GRIN lens was implanted. At 2 to 3 wks after lens implantation, a baseplate for a miniature microscope was attached at the optimal focal plane. Over 1 wk after baseplate attachment, the mice were habituated to the test environment and subjected to the behavioral experiments. (*B*, *Left*) Representative image of active dDG neurons in a wild-type mouse. The image shows the projection of the maximum relative fluorescence change (Δ*F*/*F*) of the Ca^2+^ transient among the entire set of frames from the recording of the 30-min OF experiment. Orange circles indicate the regions of interest of the recorded dDG neurons. (*B*, *Right*) Δ*F*/*F* of the Ca^2+^ signals for 15 cells. (Scale bars: 30 s [horizontal] and 5% Δ*F*/*F* [vertical]). (*C*) Color-coded spatial tuning maps, speed-tuning curves, and tuning maps of motion direction of three representative dDG neurons from wild-type mice (*Left*) and αCaMKII^+/−^ mice (*Right*) are shown. (*C*, *Top*) Spatial tuning maps showing the density of mouse locations where Ca^2+^ transients were detected. Each map is normalized to each neuron’s maximum Ca^2+^ transient rate and smoothed with a Gaussian kernel (σ = 2.0 cm). (*C*, *Middle*) Speed-tuning curves representing the mean Ca^2+^ transient rate (s^−1^) when the mouse moved at 0 to 1 cm/s, 1 to 5 cm/s, and >5 cm/s. (*C*, *Bottom*) Direction-tuning maps showing the mean Ca^2+^ transient rate when the motion direction of the mouse was in each of the eight indicated directions. Each map is normalized to the neuron’s maximum mean Ca^2+^ transient rate. North is defined as 0 radians; west is defined from 0 to π radians; and east is defined from 0 to –π radians. (*D*) Distributions of information content from Obs. neurons (color-coded curves) and those from Shuff. data (gray curves; 1,000 shuffles per cell; counts normalized by the number of shuffles) for all neurons from all animals (508 neurons from seven wild-type mice and 289 neurons from five αCaMKII^+/−^ mice). The dashed line shows the 95th percentile of the distribution of the Shuff. data, and the numbers on the lines represent the percentage of Obs. neurons exceeding this value.

By using a head-mounted miniaturized microscope, we recorded neural activity in the dDG of freely moving mice while they were traveling in an OF (Fig. 1 *A* and *B*). To minimize the tissue damage by lens implantation, we recorded the neural activities from the upper blade of the dDG. We observed that over 90% of all GCaMP-expressing neurons in the field of view were active in both wild-type and αCaMKII^+/−^ mice (*SI Appendix*, Fig. S1) and included an average 72.6 ± 9.9 dDG neurons from seven wild-type mice and an average 57.8 ± 9.6 dDG neurons from five αCaMKII^+/−^ mice into the analysis (for details, see *Detection of Ca^2+^ Transients*).

We analyzed the tuning specificities of single dDG neurons regarding mouse position, speed, and motion direction. Representative spatial tuning maps, speed-tuning curves, and tuning maps of motion direction of individual dDG neurons are shown in Fig. 1*C*. We computed information content (bits/calcium transient) (36) about each neuron’s position, speed, and motion direction, which we designated spatial, speed, and direction information, respectively. In wild-type mice, the average of spatial information of the dDG neurons was significantly larger than that expected by chance (permutation test, *P* < 10^−3^; effect size, Cohen’s *d* = 0.53; Fig. 1*D*). The distribution of the spatial information was shifted to be slightly larger than that of the shuffled (Shuff.) data (Fig. 1*D*), and the significance of spatial information for each cell varied widely from small to large (*SI Appendix*, Fig. S2), indicating that most of the dDG neurons are tuned to information about position to varying degrees. The same was also true of the amount of speed information (*P* < 10^−3^; *d* = 0.54; Fig. 1*D*). The amount of direction information was also larger than that expected by chance (*P* = 3.00 × 10^−3^; Fig. 1*D*), and the effect size was small (*d* = 0.11; Fig. 1*D*), suggesting that the activity patterns of dDG neurons are relatively weakly tuned to motion direction. Similarly, in αCaMKII^+/−^ mice, the spatial and speed information of dDG neurons was significantly greater than would be expected by chance (spatial, *P* < 10^−3^, *d* = 0.38; speed, *P* < 10^−3^, *d* = 0.58; Fig. 1*D*), but direction information was not (*P* = 0.03, *d* = 0.01; Fig. 1*D*). Thus, in both wild-type and αCaMKII^+/−^ mice, individual dDG neurons, as a whole, are significantly tuned to position and speed, but are relatively weakly tuned to motion direction. Indeed, it appears that these information types are widely distributed in the populations of dDG neurons.

### Information about Position, Speed, and Motion Direction Is Encoded in the Population Activity Patterns of dDG Neurons.

**Fig. 2.**
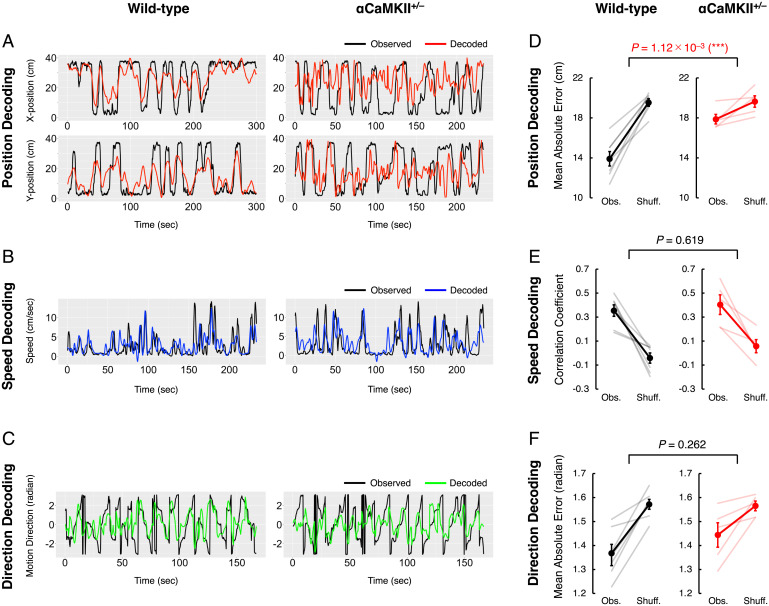
Information about position, speed, and motion direction is encoded in the population activity patterns of dDG neurons. (*A*) Representative results of position decoding from one mouse for wild-type mice (*Left*) and αCaMKII^+/−^ mice (*Right*). The mouse’s *X* and *Y* positions (cm) in the OF are shown in *A*, *Upper* and *A*, *Lower*, respectively. Black lines show the Obs. *X* and *Y* positions of the mice (Obs.), and the red lines show the positions decoded from the population activity patterns of dDG neurons (decoded). (*B*) As for *A*, but for speed instead of position. (*C*) As for *A*, but for direction instead of position. (*D*) Accuracy of position decoding in seven wild-type mice (*Left*) and five αCaMKII^+/−^ mice (*Right*). The accuracy of position decoding is reported as the mean absolute error. Black and red lines represent means, and error bars indicate the SEM. (*E*) Accuracy of speed decoding, represented as the correlation coefficient between the Obs. and decoded speeds of the mice in each time bin. (*F*) Accuracy of direction decoding. The decoding error for motion direction is computed as the mean absolute error between the Obs. and decoded motion directions in each time bin.

Next, to examine the information coding at the population level, we tested whether the mice’s position, speed, and motion direction could be decoded from the population activity of dDG neurons using machine-learning methods. Eight models were considered in preliminary analyses. Since there was no significant difference in the decoding accuracy among these models, we mainly present the results obtained using the Long Short-Term Memory (LSTM), which showed slightly higher accuracies than others (*SI Appendix*, Fig. S3, *Left*). Representative decoding results are shown together with the actual positions, speeds, and motion directions in Fig. 2 *A*–*C* (decoding accuracies for all mice are shown in *SI Appendix*, Figs. S4–S6). In wild-type mice, the decoding accuracies for position, speed, and motion direction were significantly higher than those expected by chance, which is the decoding accuracy obtained with the Shuff. data [paired *t* test, position, *t*(6) = 7.898, *P* = 2.18 × 10^−4^; speed, *t*(6) = 5.015, *P* = 2.42 × 10^−3^; motion direction, *t*(6) = 5.614, *P* = 1.36 × 10^−3^; Fig. 2 *D*–*F*, *Left*; on average, 72.6 ± 9.9 cells are used for decoding]. These results indicate that information about position, speed, and motion direction is encoded in the population activity of dDG neurons in wild-type mice.

In αCaMKII^+/−^ mice, the decoding accuracies for speed were significantly higher than those expected by chance [paired *t* test, *t*(4) = 3.965, *P* = 1.66 × 10^−2^; on average, 57.8 ± 9.6 cells are used for decoding], and those for position and motion direction were better than chance to a marginally significant degree [paired *t* test, position, *t*(4) = 2.687, *P* = 0.0549; motion direction, *t*(4) = 2.711, *P* = 0.05349]. The decoding accuracies for position were significantly lower than those of wild-type mice [unpaired *t* test, observed (Obs.)–Shuff. is compared; *t*(10) = 4.155, *P* = 1.12 × 10^−3^; Fig. 2*D*], while those for speed and motion direction were not [unpaired *t* test, Obs.–Shuff. is compared; speed, *t*(10) = 0.5571, *P* = 0.619; motion direction, *t*(10) = 1.239, *P* = 0.262; Fig. 2 *E* and *F*]. This weaker position-decoding accuracy in αCaMKII^+/−^ mice compared to wild-type mice is unlikely to be the result of using fewer neurons for decoding (*SI Appendix*, Fig. S7). Thus, in αCaMKII^+/−^ mice, information about position is selectively impaired in the dDG, whereas speed and motion-direction information is not. These results imply that, in contrast to speed and motion direction, information about position is encoded in the dDG by a different population of neurons, a different coding principle, or both (for details, see *SI Appendix*, *Position and Speed Information Are Encoded in the dDG by a Different Coding Principle*).

### Information about Position, Speed, and Motion Direction Is Independently Distributed in the Population Activity Patterns of dDG Neurons.

**Fig. 3.**
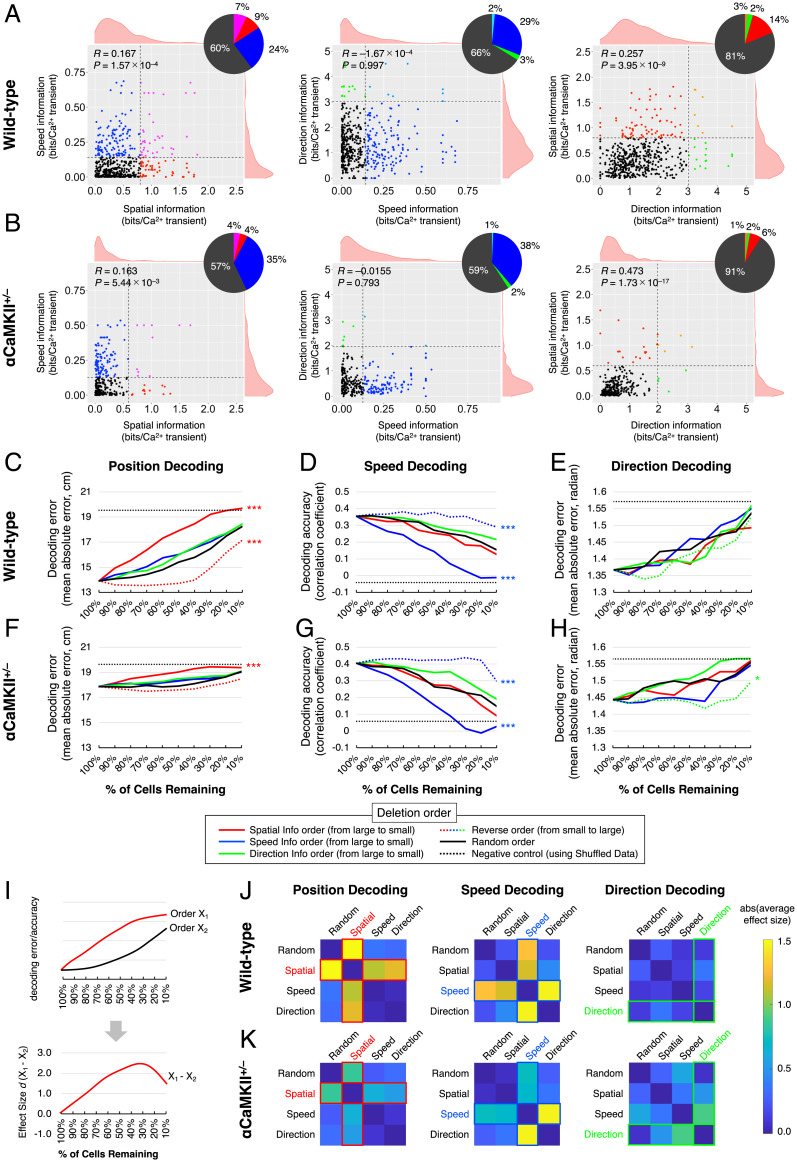
Information about position, speed, and motion direction is independently distributed in the population activity patterns of dDG neurons. (*A*, *Left*) Scatterplot showing the distribution of spatial and speed information from 508 dDG neurons from seven wild-type mice. Each dot corresponds to an individual neuron. Vertical and horizontal dashed lines indicate the 95th percentile of spatial and speed information obtained from Shuff. data (same as in Fig. 1*D*). Pie chart represents the percentage of neurons with spatial and speed information above or below this value. *R* values are correlation coefficients. (*A*, *Center* and *Right*) Scatterplots showing the distribution of speed and direction information and of direction and spatial information, respectively. (*B*) Same as for *A*, but for 289 dDG neurons from five αCaMKII^+/−^ mice. (*C*) Changes in position-decoding performance when neurons are individually deleted from the datasets. Each line shows the average data from the seven wild-type mice. Two-way ANOVA; deletion order, *F*(4, 36) = 31.46, *P* = 6.89 × 10^−22^; % of cells remaining, *F*(9, 36) = 34.64, *P* = 1.37 × 10^−41^; Tukey–Kramer post hoc tests were performed for multiple comparison, and *P* value indicates the significance of the difference between random order and others. ***P < 0.001. (*D* and *E*) Same as for *C*, but showing the changes in speed- and direction-decoding performance [speed decoding, *F*(4, 36) = 26.15, *P* = 1.30 × 10^−18^; direction decoding, *F*(4, 36) = 1.20, *P* = 0.313; Tukey–Kramer post hoc tests, vs. random order]. ***P < 0.001. (*F–H*) As for *C–E*, but showing average data from the five αCaMKII^+/−^ mice [two-way ANOVA; deletion order, position decoding, *F*(4, 36) = 8.30, *P* = 3.25 × 10^−6^, speed decoding, *F*(4, 36) = 12.36, *P* = 5.32 × 10^−9^; direction decoding, *F*(4, 36) = 4.34, *P* = 2.20 × 10^−3^; Tukey–Kramer post hoc tests were performed for multiple comparison, and *P* value indicates the significance of the difference between random order and others]. *P < 0.05; ***P < 0.001. (*I*, *Upper*) Schematic graph of two different deletion orders (*x*_1_ and *x*_2_) in decoding error/accuracy in *C–H*. (*I*, *Lower*) Effect size of decoding error/accuracy between two different deletion orders in *I*, *Upper* (*x*_1_ and *x*_2_). The effect sizes were calculated at nine different points (90 to 10% in *y* axis). (*J* and *K*) Heatmap showing the absolute value of average effect size between two different deletion orders in wild-type mice (*J*) and in αCaMKII^+/−^ mice (*K*).

Here, we investigated how each neuron encodes position, speed, and motion direction. At first, to clarify the relationship among the amount of information encoded by individual neurons for these three information types, we examined correlations between them. Scatterplots for each pair of variables (among position, speed, and direction information) show fairly uniform distributions and relatively weak correlations (spatial × speed, *R* = 0.167; speed × direction, *R* = –1.67 × 10^−4^; direction × spatial, *R* = 0.257; Fig. 3*A*). These weak correlations suggest limited correspondences between how each form of information is encoded in individual neurons. Thus, dDG neurons are diversely and independently tuned to multiple information types at the single-cell level.

The tuning specificity of a single neuron may not necessarily imply its importance in population coding. We, therefore, investigated how dDG neurons encode these multiple information types at the level of population coding. We examined whether populations of dDG neurons used for coding position, speed, and motion direction were similar to, mutually exclusive from, or independent of each other by assessing how the decoding performance changes by deleting neurons from the datasets one by one (see also *SI Appendix*, Fig. S8). In our analysis, when neurons were deleted from the dataset in order of decreasing spatial information, position-decoding accuracy decreased more rapidly than when they were deleted randomly (spatial information [info] order vs. random order, *P* = 2.29 × 10^−10^; Fig. 3*C*). The reverse was true of deletion to increase spatial information (reverse spatial info order vs. random order, *P* = 5.20 × 10^−4^; Fig. 3*C*). These results indicate that neuron populations consisting of neurons with larger spatial information tend to be more important in the population coding of information about position than those with a smaller amount of spatial information (see also *SI Appendix*, Fig. S8*A*). Furthermore, removing neurons in order to decrease speed or direction information mimicked the impacts of randomly ordered deletion on the position-decoding performance (Fig. 3*C*). These results support the notion that neuron populations with larger amounts of information about speed or motion direction are independent of those important in the coding of information about position (see also *SI Appendix*, Fig. S8*B*). Similarly, the removal of neurons in decreasing speed-information order lowered speed-decoding accuracy to a greater extent than randomly ordered deletion (speed info order vs. random order, *P* = 1.40 × 10^−8^; Fig. 3*D*); on the other hand, deletion in the reverse order had smaller impacts on speed-decoding accuracy (reverse speed info order vs. random order, *P* = 4.23 × 10^−3^; Fig. 3*D*). Removal of neurons in decreasing spatial or direction-information order did not decrease speed-decoding performance more rapidly than randomly ordered deletion (Fig. 3*D*), showing that neuron populations with larger amounts of information about position or motion direction are independent of those important in the speed-information coding. For direction decoding, there was no significant difference in the decoding accuracy for the removal of neurons in the decreasing spatial, speed, or direction-information order, the increasing direction-information order, or the random order (Fig. 3*E*). These results showed that the amount of spatial, speed, or direction information in an individual neuron is not associated with its contribution to the decoding accuracy of motion direction. Thus, neuron populations involved in coding these three types of information are not mutually exclusive or similar, but, rather, independent of each other. In addition, to quantitatively assess the degree of the independency between distributions of information about different types, we evaluated the degree of independency of the decoding error/accuracy in two different deletion orders (Fig. 3 *I*–*K*). We measured the effect size between the decoding error/accuracy in two deletion orders at nine points on the *x* axis (from 90 to 10% in percent of remaining cells; Fig. 3 *C*–*H*) and calculated the absolute values of average effect size for each pair of different deletion orders (Fig. 3*I*). In position decoding of wild-type mice, the average effect size between random order and spatial information order was 1.56 (Fig. 3*J*), indicating that information about position is preferentially encoded by neuron populations with larger spatial information, and the average effect size between random order and speed-/direction-information order was 0.35 and 0.32, respectively (Fig. 3*J*), suggesting that neuron populations with larger speed/direction information are independent of those with larger spatial information. Similarly, in speed decoding, the average effect sizes between random order and spatial, speed-, and direction-information order were 0.23, 1.27, and 0.21, respectively (Fig. 3*J*), showing that the distribution of speed information is biased to neuron populations with larger speed information and is independent of those with larger spatial/direction information. In contrast, in direction decoding, the average effect size between random order and direction-information order was 0.11, suggesting that direction information is diffusely distributed and that there is no detectable dependency among the other orders (see also *SI Appendix*, Fig. S8 *A* and *B*). Note that, as mentioned above, there is also another possibility that different information is encoded in the dDG by different coding principles (details are described in *SI Appendix*, *Supplementary Results* and Fig. S9).

### The Activity Patterns of dDG Neurons Are Tuned to Current and Future Locations to Varying Degrees.

**Fig. 4.**
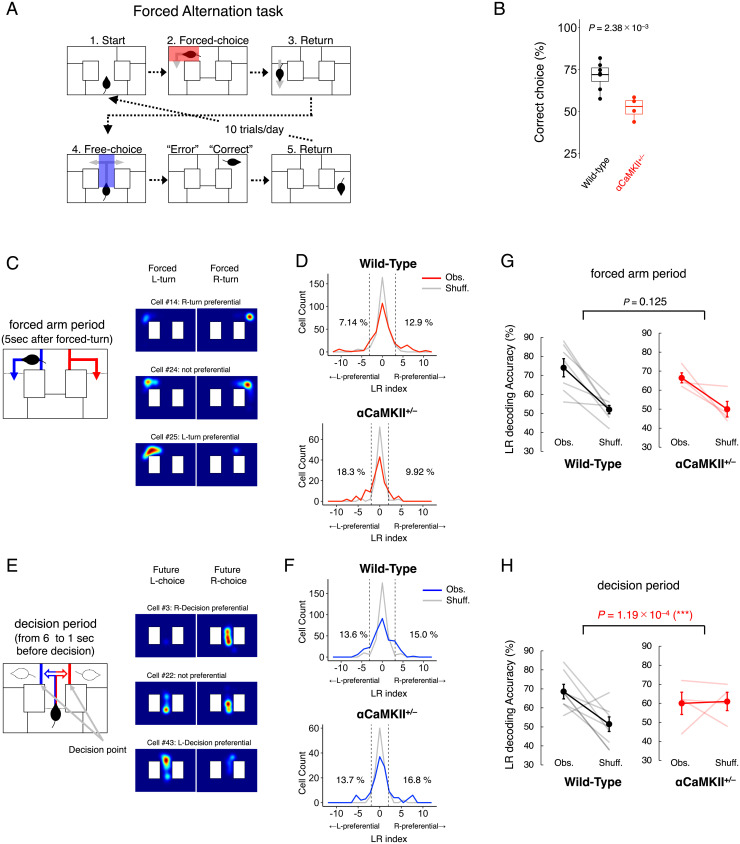
Information about current and future LR locations is encoded in the population activity patterns of dDG neurons. (*A*) Forced-alternation task in a modified T-maze test. Each trial consists of a forced-choice run followed by a free-choice run. In the forced-choice run, the mouse is forced to turn one of two randomly selected directions (1. Start → 2. Forced choice), whereas in the free-choice run, the mouse can choose to turn left or right (3. Return → 4. Free choice). This task exploits the behavioral tendency of mice to choose the arm opposite the one previously chosen. Therefore, if the direction the mouse chooses in the free-choice run is opposite to the direction presented to it in the forced-choice run, the trial is considered “correct”; otherwise, it is considered an “error.” (*B*) The correct-choice rate (%) in the forced-alternation task in seven wild-type mice and four αCaMKII^+/−^ mice. The center lines, box boundaries, and whiskers for the boxplot indicate median, upper and lower quartile, and maximum and minimum of the data. (*C*, *Left*) The forced-arm period was defined as the 5-s period after the mouse performed the forced turn. (*C*, *Right*) Color-coded cell-activity maps of three representative dDG neurons from wild-type mice. These maps show the density of mouse locations where Ca^2+^ transients were detected in the cases of an L-turn or R-turn in all 50 trials. Each map is normalized to each neuron’s maximum Ca^2+^ transient rate and smoothed with a Gaussian kernel (σ = 2.0 cm). (*D*) Distributions of LR indices calculated from the recordings of the Obs. neurons (red curve) and from Shuff. data (gray curve; 1,000 shuffles per cell; counts normalized by the number of shuffles) in the arm period, which corresponds to the 5 s following the forced turn. All 294 neurons from seven wild-type mice and 131 neurons from four αCaMKII^+/−^ mice are shown in histograms. The dashed line shows the 95th percentile of the distribution of the Shuff. data, and the numbers on the lines represent the percentage of Obs. neurons exceeding this value. (*E*, *Left*) The decision period was defined as the 6- to 1-s period prior to the free-choice turn. (*E*, *Right*) As for *C*, but for color-coded cell activity maps of representative dDG neurons in the decision period. (*F*) As for *D*, but for LR indices in decision period, which corresponds to the 6 to 1 s before the turn decision is made. (*G*) LR decoding accuracy in wild-type mice and αCaMKII^+/−^ mice from the population activity patterns of dDG neurons in the arm period. The gray (*G*, *Left*, wild-type mice) and pale red lines (*G*, *Right*, αCaMKII^+/−^ mice) show the decoding accuracy of the individual mice in each group. Black (*G*, *Left*, wild-type mice) and red (*G*, *Right*, αCaMKII^+/−^ mice) lines indicate the average decoding accuracy of all mice in each group. Error bars indicate SEM. (*H*) As for G, but for the decision period.

Next, we recorded the activity patterns of dDG neurons while the mice performed a forced-alternation task in a modified T-maze (Fig. 4*A*). For the T-maze, a different set of mice from those used for the OF was used (seven wild-type mice and four αCaMKII^+/−^ mice; for details, see *SI Appendix*, *Behavioral Experiments*). We used the T-maze test because it has been used previously to assess working memory in rodents (37) and because dysfunction in the DG is associated with reduced performance in this test (34, 35). While the OF test was used to reveal how dDG neurons represent the mouse’s current state (position, speed, and motion direction), we employed the T-maze test to examine how information is encoded in dDG neurons on where they are now and where they plan to go in the future. Wild-type mice made the correct choice (that is, during the free-choice period, they chose the arm opposite the arm that was open in the forced-choice period) 71.4% of the time, with an average of 50 sessions. In contrast, the αCaMKII^+/−^ mice showed 52.0% correct choice, on average, which is close to chance (50%) and significantly lower than the performance of the wild-type mice [*t*(9) = 4.087, *P* = 2.38 × 10^−3^; Fig. 4*B*], indicating that the αCaMKII^+/−^ mice showed deficits in this working-memory task.

Next, to elucidate the functional properties of dDG neurons in the T-maze test, we calculated the “LR index,” which takes a higher absolute value if a neuron shows differential activity patterns when the mouse is on either the left or right side of the apparatus. Here, “current location” designates the (left or right) mouse’s location at the time of recording. These neural activity patterns were taken from 5 s after the mouse performed the initial forced turn, which was designated the “forced-arm period” (Fig. 4*C*). We defined “decision period” as the period from 6 to 1 s prior to the free-choice turn (Fig. 4*E*) and “future location” as the (left or right) location of the mouse after the decision period. The left or right during the decision period is defined by that of the future location—i.e., left or right that the mouse will choose after the free-choice turn. We used the neural activity during the decision period to calculate the LR index and decode the future LR location. Note that in this period, the future location did not significantly correlate with the mouse’s physical location, speed, and motion direction of the mouse (*SI Appendix*, Fig. S10 *G–I*). In the wild-type mice, the absolute values of the LR indices of neurons during the forced-arm period were significantly higher than those expected by chance (permutation test, *P* < 10^−3^; effect size, Cohen’s *d* = 0.43). The distribution of LR indices of neurons was shifted to be slightly larger in absolute values than that of the Shuff. data (Fig. 4 *D*, *Upper*), showing that the activity patterns of dDG neurons are tuned to left or right of the current location to varying degrees. The same was also true of the LR indices of neurons during the decision period in wild-type mice (*P* < 10^−3^, *d* = 0.52; Fig. 4 *F*, *Upper*) and of those in the forced-arm and decision period in αCaMKII^+/−^ mice (forced-arm period, *P* < 10^−3^, *d* = 0.51; decision period, *P* < 10^−3^, *d* = 0.54; Fig. 4 *D* and *F*, *Lower*). These results showed that the activity patterns of dDG neurons of wild-type and αCaMKII^+/−^ mice are significantly tuned to both current and future LR locations.

### Information about Current and Future LR Location Is Encoded in the Population Activity Patterns of dDG Neurons.

Next, we trained a binary decoder on the population activity data obtained from dDG neurons and tested whether it could estimate the LR location of the mice in the forced-arm and decision periods. In wild-type mice, we were able to decode the current LR location of the mice in the forced-arm period from the population activity patterns of dDG neurons more accurately than those expected by chance [paired *t* test, *t*(6) = 4.621, *P* = 3.61 × 10^−3^; Fig. 4 *G*, *Left*]. We were also able to estimate the future LR location of mice from population activity patterns in the decision period more accurately than that expected by chance [*t*(6) = 2.929, *P* = 0.0263; Fig. 4 *H*, *Left*]. These results suggest that information about the current and future LR location may be encoded in dDG neuron activity patterns obtained in the current and decision period, respectively. Meanwhile, in αCaMKII^+/−^ mice, the decoding accuracy of the current LR location in the forced-arm period was more accurate than that expected by chance [paired *t* test, *t*(3) = 2.819, *P* = 0.0186; Fig. 4 *G*, *Right*] and not significantly different from that of wild-type mice [unpaired *t* test, Obs.–Shuff. is compared, *t*(9) = 1.367, *P* = 0.125; Fig. 4*G*], indicating that information about current LR location is encoded in dDG neuron activity patterns of αCaMKII^+/−^ mice. The successful decoding of the current LR location in the T-maze in αCaMKII^+/−^ mice seems to contradict the results that position in the OF test was not accurately decoded from the neuronal activities in the DG of these mice (Fig. 2 *A* and *D*). This may be because the defined area of the current LR location (5 s after the turn, ∼10 cm) is much larger than the resolution of position decoding in the OF. Indeed, the position-decoding performance of αCaMKII^+/−^ mice in the T-maze is worse than the wild-type (*SI Appendix*, Fig. S11), as was seen in the OF, while the decoding performance of the current LR of the mutants was better than the chance level. We also aimed to estimate the future LR location of αCaMKII^+/−^ mice from the population activity of the decision period, but its accuracy was not higher than that expected by chance [paired *t* test, *t*(3) = 0.1325, *P* = 0.903; Fig. 4 *H*, *Right*] and was significantly lower than that of wild-type mice [unpaired *t* test, Obs.–Shuff. is compared, *t*(9) = 8.401, *P* = 1.19 × 10^−4^; Fig. 4*H*], suggesting that future LR choice is not accurately represented in dDG neurons in αCaMKII^+/−^ mice. Thus, the neural representation of future location is selectively impaired in the dDG of αCaMKII^+/−^ mice, whereas that of the current LR location in the T-maze is not, implying that current and future locations in the T-maze may be independently encoded among the populations of dDG neurons.

### Information about Current and Future LR Locations Is Widely Distributed in the Population Activity Patterns of dDG Neurons.

**Fig. 5.**
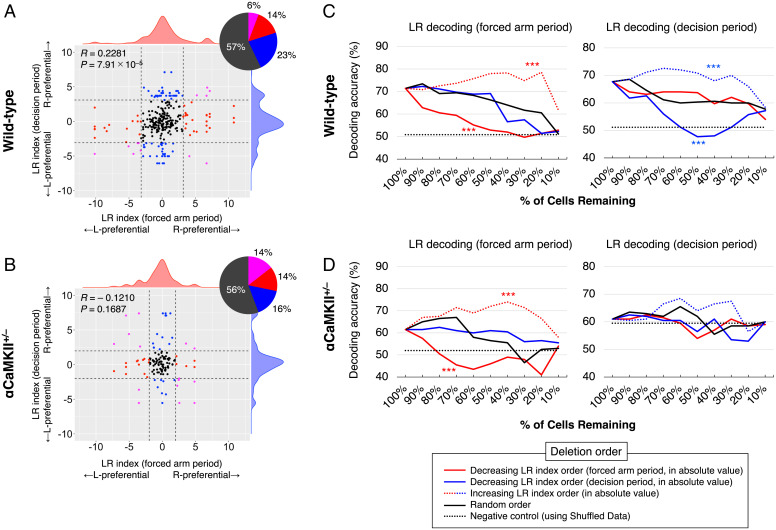
Information about current and future LR locations is widely distributed in the population activity patterns of dDG neurons. (*A* and *B*) Scatterplot showing the distribution of the LR indices of dDG neurons in the forced-arm and decision periods. Each dot corresponds to an individual neuron. A total of 294 neurons from seven wild-type mice (*A*) and 131 neurons from four αCaMKII^+/−^ mice (*B*) are shown. The horizontal and vertical axes indicate the LR indices from the forced-arm and decision periods, respectively. Vertical and horizontal dashed lines indicate the 95th percentile of LR indices from the forced-arm and decision periods obtained from Shuff. data (same as in Fig. 4 *C* and *E*). Pie charts represent the percentage of neurons with LR indices above or below this value. *R* values are correlation coefficients. (*C* and *D*) Changes in LR decoding performance (*Left*, arm period; *Right*, decision period) when neurons were individually deleted from the datasets for wild-type mice (*C*) and αCaMKII^+/−^ mice (*D*). The horizontal axis represents the percentage of remaining neurons that are used for population decoding after neurons are deleted. The vertical axis shows the LR decoding accuracy (%) in the arm period (*Left*) and the decision period (*Right*). Each line shows the average of seven wild-type mice (*C*) and four αCaMKII^+/−^ mice (*D*). Two-way ANOVA, arm period of wild-type mice, *F*(3, 27) = 34.92, *P* = 9.14 × 10^−19^; decision period of wild-type mice, *F*(3, 27) = 12.00, *P* = 2.36 × 10^−7^; arm period of αCaMKII^+/−^ mice, *F*(3, 27) = 16.98, *P* = 2.95 × 10^−9^; decision period of αCaMKII^+/−^ mice, *F*(3, 27) = 1.39, *P* = 0.249; Tukey–Kramer post hoc tests were performed for multiple comparison, and *P* value indicates the significance of the difference between random order and others. ***P < 0.001.

Next, we investigated the relationship between LR indices in the forced-arm and decision periods of each dDG neuron. The scatterplot of the LR indices of the dDG neurons obtained in the forced-arm and decision periods of the wild-type mice showed a small, but statistically significant, correlation (*R* = 0.2281, *P* = 7.91 × 10^−5^; Fig. 5*A*). Meanwhile, in αCaMKII^+/−^ mice, the LR indices of the neurons obtained in the forced-arm and decision periods showed a uniform distribution, and these indices were not significantly correlated (*R* = –0.1210, *P* = 0.1687; Fig. 5*B*). Thus, the LR tunings of dDG neurons about current and future LR locations are weakly correlated in wild-type mice and largely independent of each other in αCaMKII^+/−^ mice. These correlations suggest that there is a significant, but weak, relationship between LR tunings of individual dDG neurons during the forced-arm and decision period in wild-type mice (see also *SI Appendix*, Fig. S12).

To examine how information about the current and future LR locations is distributed in the population activity patterns of dDG neurons, we assessed how the LR decoding accuracy in the forced-arm and decision periods changed when neurons were removed from the datasets one by one. In the data collected in wild-type mice during the arm period, upon deletion of neurons in order of decreasing LR index for the forced-arm period, the LR decoding accuracy of the forced-arm period decreased more rapidly than it did upon randomly ordered deletion (decreasing LR index [in forced-arm period] order vs. randomly ordered, *P* = 1.54 × 10^−6^; Fig. 5 *C*, *Left*); deletion of neurons in order of increasing LR index had smaller impacts on the LR decoding accuracy than randomly ordered deletion (increasing LR index [in forced-arm period] order vs. randomly ordered, *P* = 1.12 × 10^−5^; Fig. 5 *C*, *Left*). However, the removal of neurons in order of increasing LR index for the decision period did not decrease decoding accuracy more rapidly than randomly ordered deletion (Fig. 5 *C*, *Left*). Meanwhile, for LR decoding in the decision period, the deletion of neurons in order of decreasing LR index for the decision period significantly decreased the decoding accuracies (decreasing LR index [in decision period] order vs. randomly ordered, *P* = 1.92 × 10^−2^; Fig. 5 *C*, *Right*), and reverse order deletion showed smaller decreases than that by random order deletion (increasing LR index [in decision period] order vs. randomly ordered, *P* = 1.27 × 10^−2^; Fig. 5 *C*, *Right*). On the other hand, removing neurons in order of decreasing LR index for the forced-arm period did not have much impact on LR decoding performance, similar to the impact of randomly ordered deletion (Fig. 5 *C*, *Right*). These results suggest that neuron population involved in coding current location is independent from that of future location and vice versa. Thus, information about the current and future location is expected to be independently distributed in the population activity patterns of dDG neurons.

## Discussion

In this study, we demonstrated that multiple types of information (position, speed, and motion direction in an OF, as well as current and future left or right location in a T-maze) were represented in the population activity patterns of dDG neurons (Figs. 2 and 4). These multiple types of information were encoded by overlapping, but independent, populations of dDG neurons (Figs. 3 and 5). Furthermore, in αCaMKII^+/−^ mice, which present deficits in spatial remote and working memory, the neural representation of information about position in the OF and future LR location in the T-maze was selectively impaired, supporting the notion that different types of information are independently distributed among dDG neurons (Figs. 2 and 4).

The accurate decoding of position (in the OF) and current LR location (in the T-maze) in wild-type mice indicates that the DG is involved in place coding, which is consistent with previous studies (3–6). Additionally, the successful decoding of speed and motion direction in the OF and future location in the T-maze in this study indicates that the dDG is also involved in the neural representation of these types of information. It is of interest that the decoding in our study was accurate, despite the fact that DG neurons are generally thought to have a lower range of firing frequencies compared to those of most other brain regions (25). In our data, the average recorded Ca^2+^ transient frequency was quite low, ∼0.02 Hz, comparable to that of granule cells (1 to 2 per min) reported by the previous imaging study (6). Our results thus suggest that the dDG neurons may be involved in information coding with their low-frequency activity patterns.

We found that the populations of DG neurons cooperate to encode information about position, speed, and motion direction in an OF, indicating that these types of information are diffusely distributed among dDG neurons, which is consistent with a recent report (7). In this report, the authors proposed that the interpretability of individual neurons is not necessarily important for their contribution to information coding based on the weak correlation between an index of spatial information and the “importance index,” a measure of the contribution of each neuron to decoding. In contrast with their conclusion, our results indicate that dDG neurons with a larger amount of information are more important for decoding position and speed in an OF than those with smaller amounts of information (Fig. 3*C* and *SI Appendix*, Fig. S8*A*). This discrepancy may be due to the difference in the methods of evaluating the importance of cells in population decoding. While the importance index evaluates the contribution of a single cell to decoding, our method evaluates the impacts of neuron deletion on decoding performance when cell populations with larger or smaller information content are deleted (Fig. 3*C* and *SI Appendix*, Fig. S8*A*). Since the contribution of a single DG neuron to population coding is quite small, it might be difficult to demonstrate the association between the importance of a single neuron in decoding and the amount of information content. For example, in our analysis, the deletion of the top 1% of neurons does not show clear differences in the decoding accuracies among deletion orders (Fig. 3*C*), but when 30 to 60% are deleted, the difference becomes much clearer (Fig. 3*C* and *SI Appendix*, Fig. S8*A*). Thus, by evaluating the impacts of neuron deletion on decoding performance, we can see a significant difference in the contribution to the population coding between neurons with larger and smaller information content, which would not be evident from the single-cell-level analysis.

We identified redundancy in the distribution of some types of information in the dDG; our results indicate that only the top 30 to 40% of all neurons were sufficient for decoding position and speed with maximum accuracy (Fig. 3*C*, red dotted line in *Left* and blue dotted line in *Center*). On the other hand, ∼70 to 80% of all the recorded neurons can contribute to position or speed decoding (Fig. 3*C*, solid red line in *Left* and solid blue line in *Center*). The neurons with an intermediate information content, which are not needed for population encoding when the neurons with the largest information content are spared, can contribute to the decoding accuracy when these top-class neurons were deleted, further demonstrating the redundancy of information encoding in the individually recorded neurons. In our study, we recorded 70 to 80 neurons per mouse, and the position-decoding accuracy was significantly higher than the chance level. The previous study using more than 300 neurons achieved more accurate decoding performance than ours (7). Considering that there are ∼500,000 neurons in the entire DG (38), there seems to be far more redundancy in the entire DG than in the population that we recorded. Generally, the number of states that a neural circuit can represent is thought to depend on the number of neurons and the dynamic range of firing frequencies of the neurons (25). Given this assumption, we propose that the redundant encodings of information in a large number of infrequently firing neurons allows the DG to represent a large number of behavioral states in its population activity patterns.

Remarkably, we also discovered that some of the neurons of the DG demonstrate mixed selectivity (Figs. 3 and 5)—i.e., each of these neurons is involved in encoding multiple types of information. Mixed selectivity of neurons has also been reported in the hippocampus (7) and other brain regions, including the medial EC, in which some neurons can encode position, speed, and head direction (39), and the parietal and frontal cortexes, in which some neurons can encode multiple behavioral or task-relevant variables (40, 41). Mixed selectivity of neurons is thought to be essential for representing many independent variables in the population activity patterns of a limited number of neurons (40, 41). There could potentially be two different possible models of tuning patterns for multiple variables in a group of neurons with or without mixed selectivity. One is that a population of neurons with large information of a variable also has a large amount of information of another variable (mixed selective and dependent; *SI Appendix*, Fig. S8 *B*, *i*). Another is that neurons with large information about a variable have a smaller amount of information about another variable (mutually exclusive; *SI Appendix*, Fig. S8 *B*, *ii*). The other is that the amount of information of a variable in a population of neurons is independent of those of other variables (mixed selective and independent; *SI Appendix*, Fig. S8 *B*, *iii*). If the distribution of these information types is in a pattern shown in (*i*), the place-coding cells would overlap with speed-coding cells. Then, the number of cells that can be used for place-coding would be limited to the number of speed-coding cells. In case (*ii*), the place-coding cells would be relegated to non-speed-coding cells. In case (*iii*), the distribution of place-coding cells is not constrained by that of speed-coding cells. In order to maximize the number of neurons that can be used to represent a certain type of information, its information needs to be distributed as independently as possible from others, as in (*iii*). In our study, by quantitatively evaluating the importance of neurons involved in decoding, we found that multiple types of information are independently distributed in the dDG not only at the level of a single neuron, but also at the level of population coding (Figs. 3 *A* and *C* and 5 *A* and *C*), as shown in (*iii*). DG has been hypothesized to be specialized for pattern separation (42), and the number of states that a neural circuit can represent is thought to be relatively larger than other regions (25). In addition, DG has been reported to encode many types of information (7, 23, 43). Considering these aspects of DG that represent a large number of states for many types of information simultaneously, it makes sense that different types of information were distributed in DG in a mixed and independent manner. Together with their diffuseness and redundancy, the mixed selectivity and independence of dDG neurons seem to allow the DG not only to express a large number of states for each type of information, but also to express these states for multiple types of information. These features may be more pronounced in DG that is hypothesized to be specialized for pattern separation than in other brain regions. Previous studies have also reported mixed selectivity of neurons in other brain regions (7, 39–41), but little has been known about the independency of coding patterns for multiple types of information in those regions. Future studies are needed to determine if the independency as seen in DG exists in other brain regions.

Mutations of αCaMKII have been reported to cause intellectual disability accompanied by epilepsy, abnormal emotional and affective behavior, and/or autistic features (44–47), and the gene has also been implicated in autism (48) and bipolar disorder (31, 49). αCaMKII^+/−^ mice have an endophenotype called iDG, in which the neurons in the DG are in a pseudoimmature status (31, 34). The phenomenon similar to their iDG can be seen in other mouse models of neuropsychiatric disorders and human patients (35, 50). One of the interesting results obtained from the αCaMKII^+/−^ mice was that the decoding accuracy of position in the OF was selectively impaired in the dDG, while those of speed and direction were not. This selective impairment of information about position in the DG of αCaMKII^+/−^ mice may be attributed to disorganized activity patterns of neurons (see also *SI Appendix*, *Position and Speed Information Are Encoded in the dDG by a Different Coding Principle*), and this may also be seen in animal models and human patients with the similar iDG phenotype. Future studies on the mechanism underlying the selective impairment of information in αCaMKII^+/−^ mice and other model mice that show similar phenotypes would contribute to understanding the pathophysiology of neuropsychiatric disorders that share this phenotype.

In conclusion, our findings suggest that multiple types of information are diffusely, redundantly, mixed-selectively, and independently encoded in the population activity patterns of dDG neurons. These features of information coding may be the basis on which the DG is involved in the processing and integration of a variety of information. Future studies are needed to determine whether information about other functions of the DG (e.g., episodic memory, object recognition, and odor-information processing) is similarly encoded in the population activity patterns of neurons and how this encoding is altered in disease states (e.g., amnesia, Alzheimer's disease, and schizophrenia).

## Materials and Methods

### Animals.

All experimental protocols were approved by the Institutional Animal Care and Use Committee of Fujita Health University. Adult male C57BL/6J mice and αCaMKII^+/−^ mice were obtained from Jackson Laboratories and were backcrossed to C57BL/6J mice (Charles River) for at least 19 generations. They were used for experiments at 50 ± 4.4 wk of age. The mice were housed one per cage in a room with a 12-h light/dark cycle (lights on at 7:00 AM, off at 7:00 PM) with access to food and water ad libitum. The room temperature was maintained at 23 ± 2 °C. All experiments were conducted during the light period.

### Surgeries.

#### Viral delivery of Ca^2+^ sensor.

For delivery of a fluorescent Ca^2+^ sensor into dDG neurons, adeno-associated virus (AAV) carrying the GCaMP6f vector was injected into the DG of the dorsal hippocampus in adult mice by a conventional method (right hemisphere; 2.0 mm posterior to bregma [AP], 1.0 mm lateral to midline [ML], and 2.0 mm ventral to bregma [DV]) (51). In brief, adult mice >8 wk old were first anesthetized with 1.5 to 3% isoflurane at an oxygen flow rate of 1 L/min. The head fur was shaved, and the incision site was sterilized with 70% ethanol prior to the surgical procedure. The mice were then mounted on a stereotaxic device (catalog no. 51730D, Stoelting), and a heat pad (catalog no. BWT-100A, BRC) was placed underneath each mouse to maintain body temperature at 37 °C. After the scalp was incised and pulled aside, a 2-mm-diameter craniotomy was created with a surgical drill on the skull above the injection site. Through a glass-capillary injection pipette (25-µm inner-diameter tip), 1 μL of AAV5-Syn-GCaMP6f-WPRE-SV40 (titer 2.8 × 10^13^ GC/mL; gift from Douglas Kim (Janelia Research Campus, Howard Hughes Medical Institute, Ashburn, VA, USA) and the GENIE Project; Addgene viral preparation #100837-AAV5) (52) was injected by using a microinjection pump (Nanoliter 2010, WPI). After the injection, the scalp was sutured and treated with povidone–iodine. DG consists of granule cells (∼90%) and contains other types of excitatory neurons, such as mossy cells in hilus and interneurons (38). GCaMP6f expression is driven by the Syn-promoter; however, it is not cell-type-specific. Therefore, its expression is expected to be induced not only in granule cells, but also in mossy cells.

#### GRIN lens implantation.

At least 2 wk after the viral injection, a gradient refractive index (GRIN) lens (Inscopix catalog no. 1050-002202; 1 mm diameter; 4.0 mm length) was implanted into each mouse. The mouse was mounted on the stereotaxic device as described above, and the scalp was removed. After exposure of the skull and removal of the overlying connective tissue, we made a cranial hole that was slightly larger than the diameter of the GRIN lens. To make a “pretrack” for the GRIN-lens insertion, we made an ∼2-mm-wide incision in the exposed cortex down to 1 mm from the brain surface and then slowly embedded the lens into the dDG (AP: −2.0 mm; ML: +1.0 mm; DV: −1.75 mm). After temporarily immobilizing the lens with an ultraviolet-curable resin (Primefil, Tokuyama Dental), the lens and a stainless frame (CF-10, Narishige) were fixed to the exposed skull by using dental cement mixed with black dye (Sudan Black, Sigma). The exposed tip of the GRIN lens was covered with dental silicone (Dent Silicone-V, Shofu). After lens implantation, analgesic and anti-inflammatory agents (Flunixin, 2 mg/kg; Fujita) and antibiotics (Tribrissen, 0.12 mL/kg; Kyoritsu Seiyaku) were injected intraperitoneally. After each experiment, we confirmed that the GRIN lens was implanted in the upper side of the granule-cell layer, where most neurons are GCs, by histological analysis (*SI Appendix*, Fig. S13).

#### Baseplate attachment.

At least 2 wk after GRIN-lens implantation, the baseplate of a miniature microendoscope (nVista 2.0, Inscopix) was attached over the GRIN lens by a conventional method (27). Briefly, the mice were anesthetized and mounted onto the stereotaxic device described above, and a baseplate attached to the miniature microscope was placed on the GRIN lens by using Gripper (Inscopix). The optimal location was determined by monitoring the fluorescent images of GCaMP-expressing neurons (where the largest number of neurons were in focus), and the baseplate was fixed with dental resin cement (Super-Bond C&B, Sun Medical) at this position. In cases where we failed to identify neurons at this stage (usually due to the failure of GRIN-lens implantation), the baseplate was not attached, and such mice were excluded from the experiment. After baseplate attachment, a baseplate cover was placed on the baseplate until Ca^2+^ imaging was performed.

### Ca^2+^ Imaging in Freely Moving Mice.

Ca^2+^ imaging of DG neurons was performed while the mice freely traveled in an OF or T-maze, each on a different day. Each different group of mice was used for an OF or T-maze. Prior to Ca^2+^ imaging, the mice were lightly anesthetized, and a miniature microscope (nVista 2.0, Inscopix) was mounted onto the baseplate of the mice. The mice were then placed back in the home cages and then transferred to a sound-proof behavioral experiment room. At least 30 min after recovery from anesthesia, the mice were subjected to OF or T-maze tests, while the Ca^2+^ signals of their DG neurons were obtained at a 3-Hz sampling rate with 1,440- × 1,080-pixel resolution. We applied 475/10-nm LED light for the excitation of GCaMP6f fluorescence (∼0.24 mW/mm^2^ at the bottom of the GRIN lens).

### Detection of Ca^2+^ Transients

To extract the activity patterns of individual DG neurons from the obtained fluorescent images, we used Inscopix Data Processing Software (IDPS). Briefly, the data files of the raw sequential fluorescent images obtained by nVista (Inscopix) were imported to IDPS, and motion correction was applied. The instantaneous fluorescence of the motion-corrected images was then normalized by its average fluorescence over the entire recording period, producing fluorescence-change ratio (Δ*F*/*F*) images. Then, individual DG neurons were identified by automated principal component analysis (PCA)/independent component analysis (ICA) segmentation of activity traces. For PCA/ICA segmentation, default parameters, whose values were confirmed to work well across most of the neuronal activity patterns in the cortex and CA1 of the hippocampus, were used (number of independent components, 120; number of principal components, 150; ICA max iterations, 100; ICA random seed, 0; ICA convergence threshold, 0.00001; block size, 1,000; ICA unmixing dimension, spatial; ICA temporal weights, 0.00; arbitrary units within IDPS). After cell identification, longitudinal cell registration was performed to align cell maps across multiple sessions, as necessary. Finally, the temporal pattern of each the timings of DG neuron’s activity was identified by an event-detection algorithm available in IDPS (event threshold factor, four median absolute deviations; event shortest decay time, 0.2 s), and the temporal pattern of activity was expressed as a time series of binarized signals for further analysis.

For details of animals, experimental procedures, and data analysis, see *SI Appendix*, *SI Methods*.

## Supplementary Material

Supplementary File

## Data Availability

The data analysis was performed by using custom code written in MATLAB (MathWorks, R2018a) and Python (Version 2.7.12). For decoding analysis by machine learning, we obtained Python code available at GitHub (https://github.com/KordingLab/Neural_Decoding) [used in Glaser et al. (53)] and modified them for our purpose. Raw imaging data in this study have been uploaded at the Systems Science Biological Dynamics repository (ssbd.qbic.riken.jp/set/20200603) (54). Processed imaging data, behavioral data, and codes in this study are available at GitHub (https://github.com/tmurano) (55).
