# Supplementary material for: Multiple types of navigational information are independently encoded in the population activities of the dentate gyrus neurons
Source: Proc Natl Acad Sci U S A. 2022 Aug 5;119(32):e2106830119. doi: 10.1073/pnas.2106830119 (PMC9371651; doi:10.1073/pnas.2106830119)
Supplement: Supplementary File [file pnas.2106830119.sapp.pdf]

Supplementary Figures

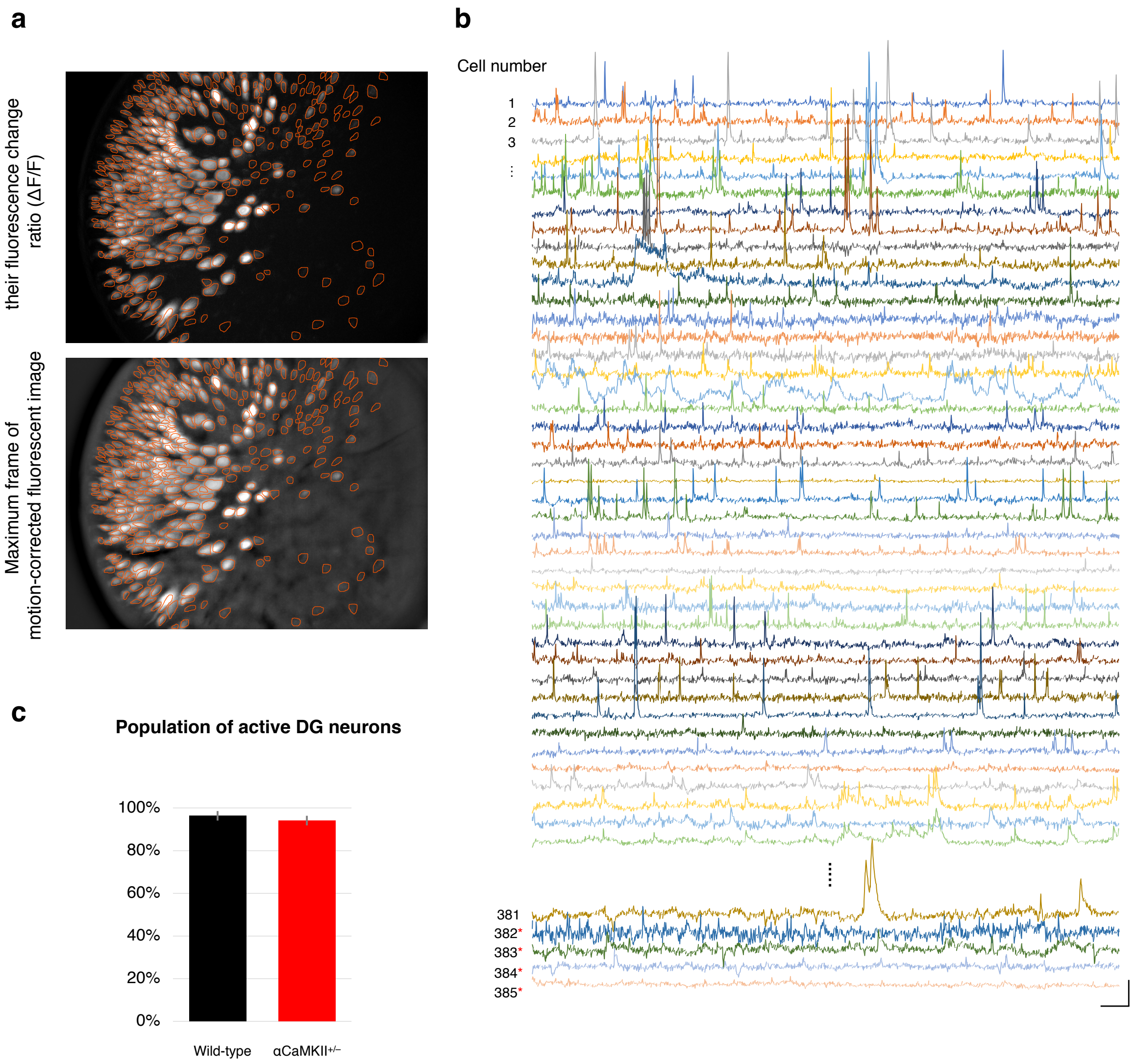

Figure S1. Proportion of active DG neurons.

a, Fluorescence change ratio (ΔF/F) image (upper panel) and maximum frame of motion-corrected (MC) fluorescent image (lower panel) of a representative wild-type mouse. Orange circles indicate the ROI of cells from the maximum projection image.

b, Representative Ca^{2+} signals (ΔF/F) of individual dDG neurons, indicated by the regions of interest (ROIs) in (a). Fifty representative cells of a total of 384 cells are shown, and red asterisks indicate GCaMP-expressing neurons whose activity was not detected in the 30 min recording. Scale bars: 30 sec (horizontal) and 5% ΔF/F (vertical).

c, The proportion of dDG neurons in the field of view that were active. The number of neurons demonstrating GCaMP expression was manually counted from the frame with maximum MC fluorescence, and the number of neurons with calcium transients (active neurons) was counted from their fluorescence change ratio ($\Delta F/F$) images. Bar graphs show the average proportion of active dDG neurons. Error bars indicate SEM.

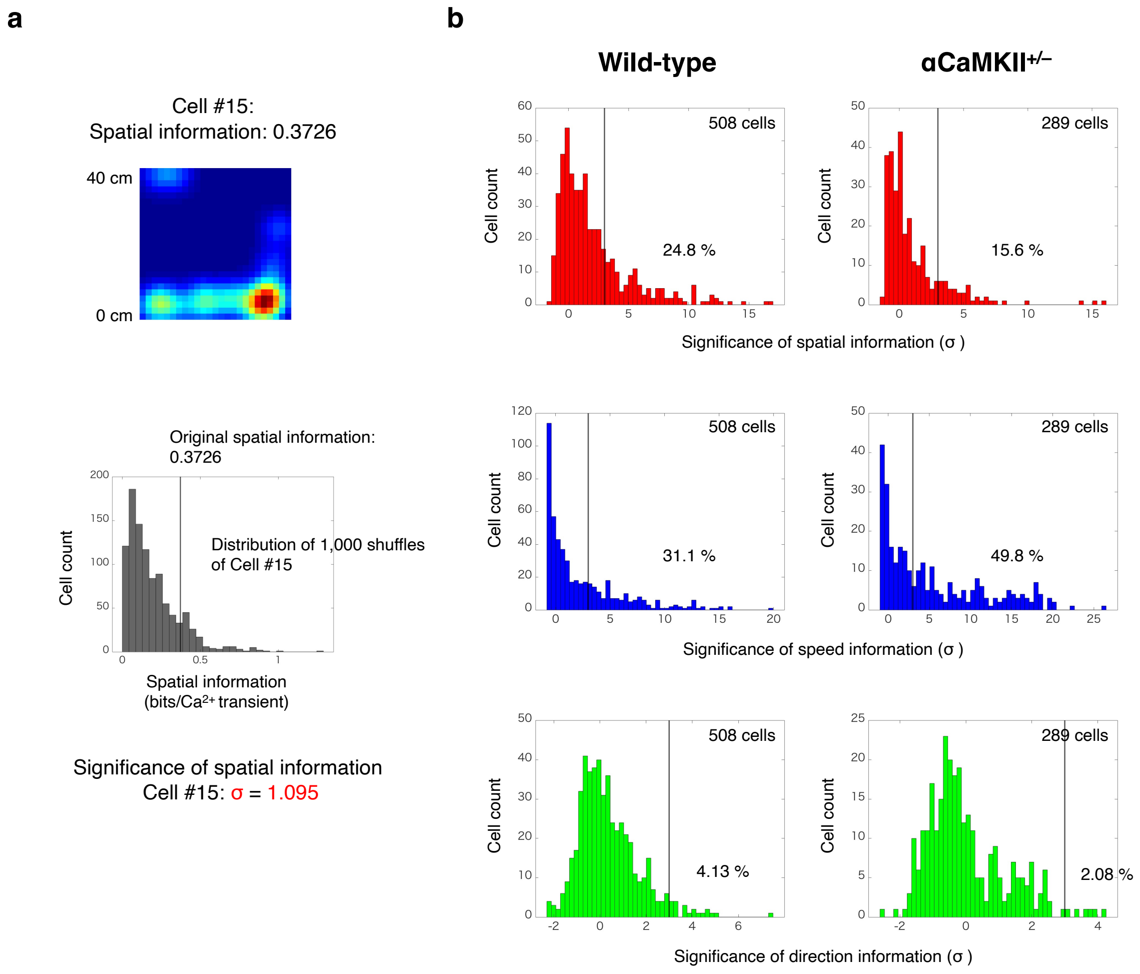

Figure S2. Significance of spatial, speed, and direction information of each dDG neuron.

a, Top: Colour-coded spatial tuning map of representative dDG neuron (Cell #15). Bottom: Distribution of spatial information from 1,000 shuffled data generated from Cell #15. The vertical black line shows the original spatial information for Cell #15. Significance of spatial information of the cell is calculated from the value of the original spatial information in the normalized distribution of shuffled data.

b, Distribution of significance of spatial, speed, and direction information for all neurons from all animals (508 neurons from 7 wild-type and 289 neurons from 5 αCaMKII$^{+/-}$ mice). The vertical black line indicates where the significance of information content (σ) is 3, and the numbers on the lines represent the percentage of neurons exceeding this value.

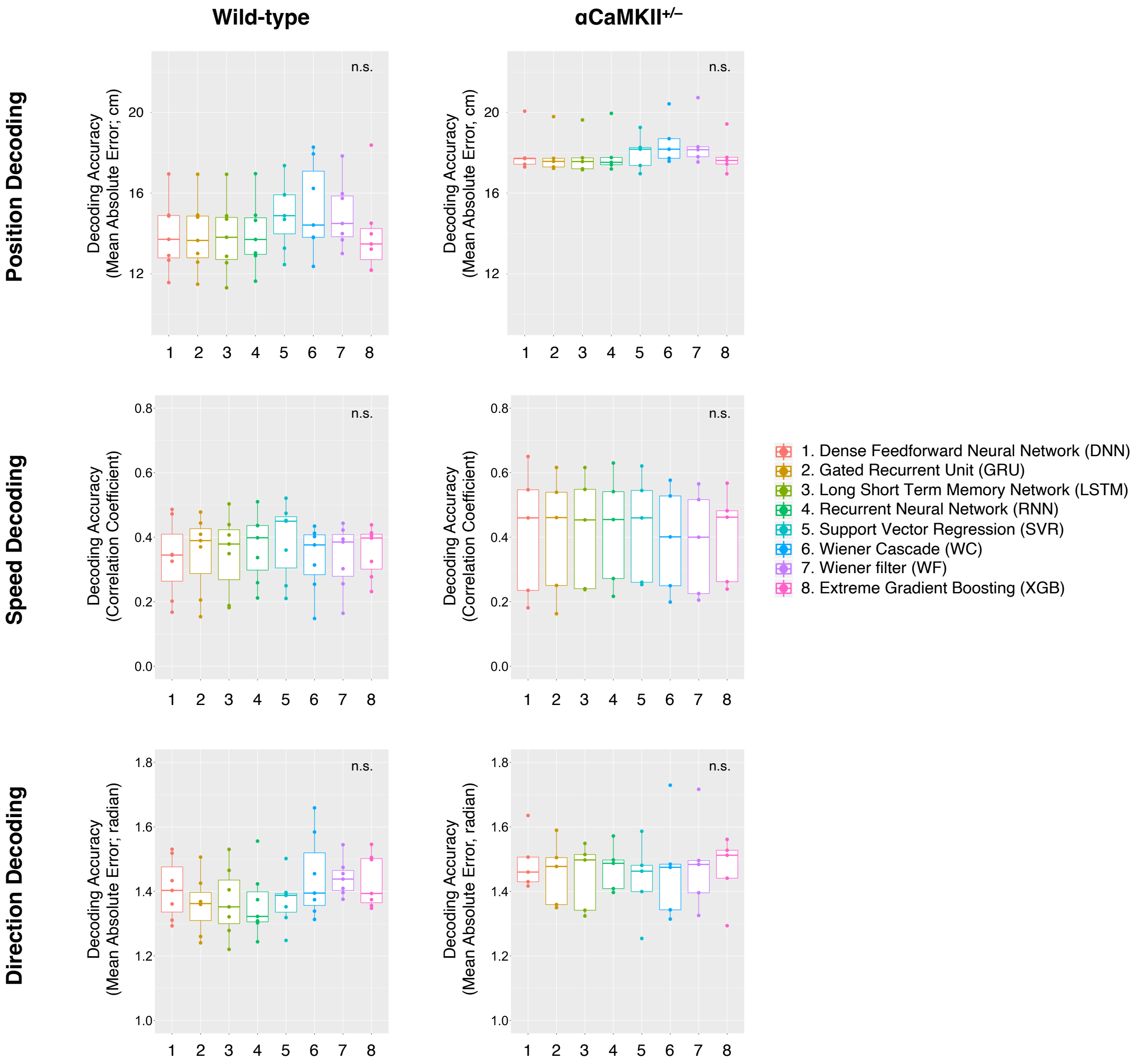

Figure S3. Machine learning methods used to decode position, speed, and motion direction.

We tested 8 different machine learning methods for separately decoding the position, speed, and motion direction in mice in the open field test (as listed in the figure). Overall, there was no significant difference in the decoding accuracies among these models.

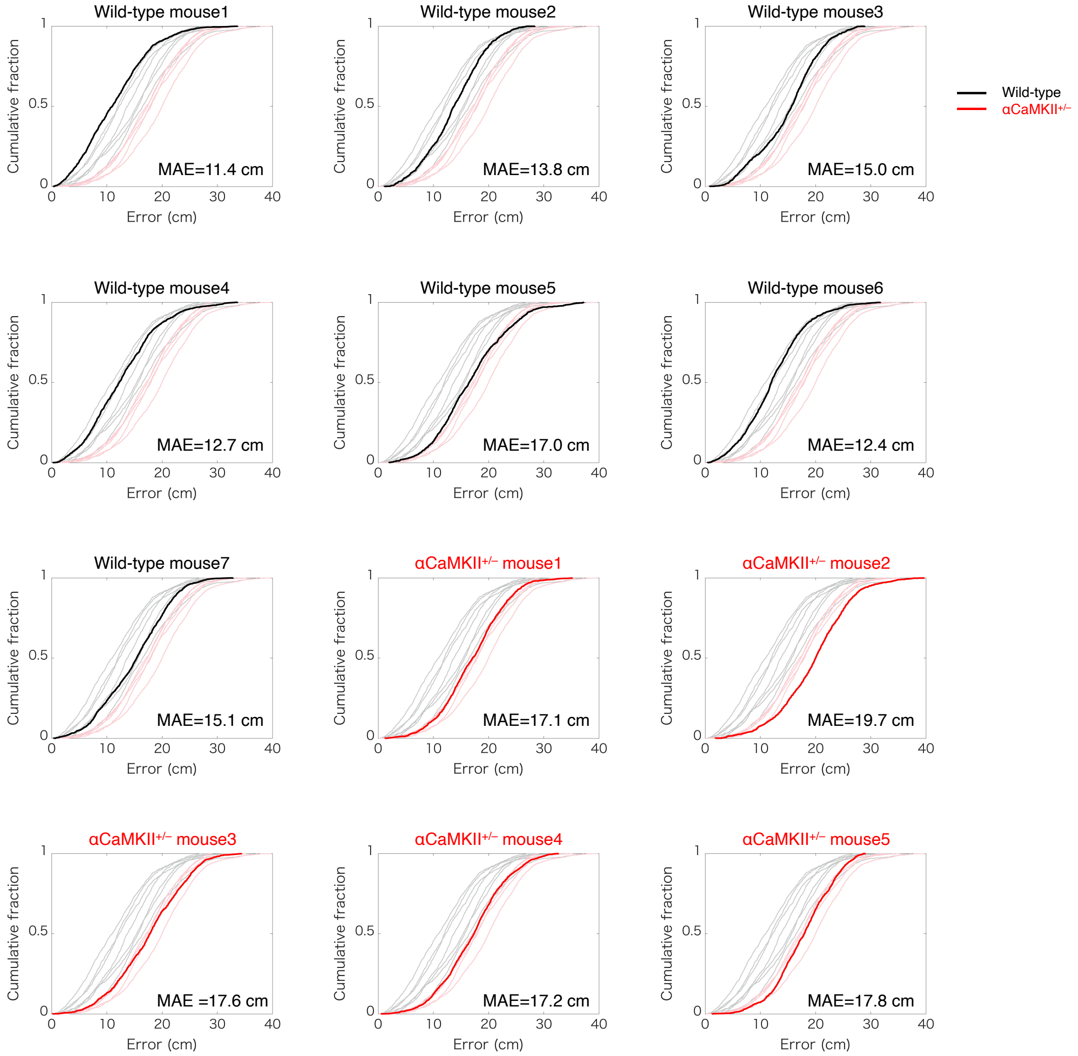

Figure S4. Position decoding error in the open field.

Cumulative distribution functions (CDFs) of the position decoding error. The results from all the mice (black; 7 wild-type mice, red; 5 αCaMKII$^{+/-}$ mice) are shown. Mean absolute errors (MAE, same as in Figure 2d) of each mouse are also shown in each figure. Note that all of the decoding errors of wild-type mice were smaller than those of αCaMKII$^{+/-}$mice.

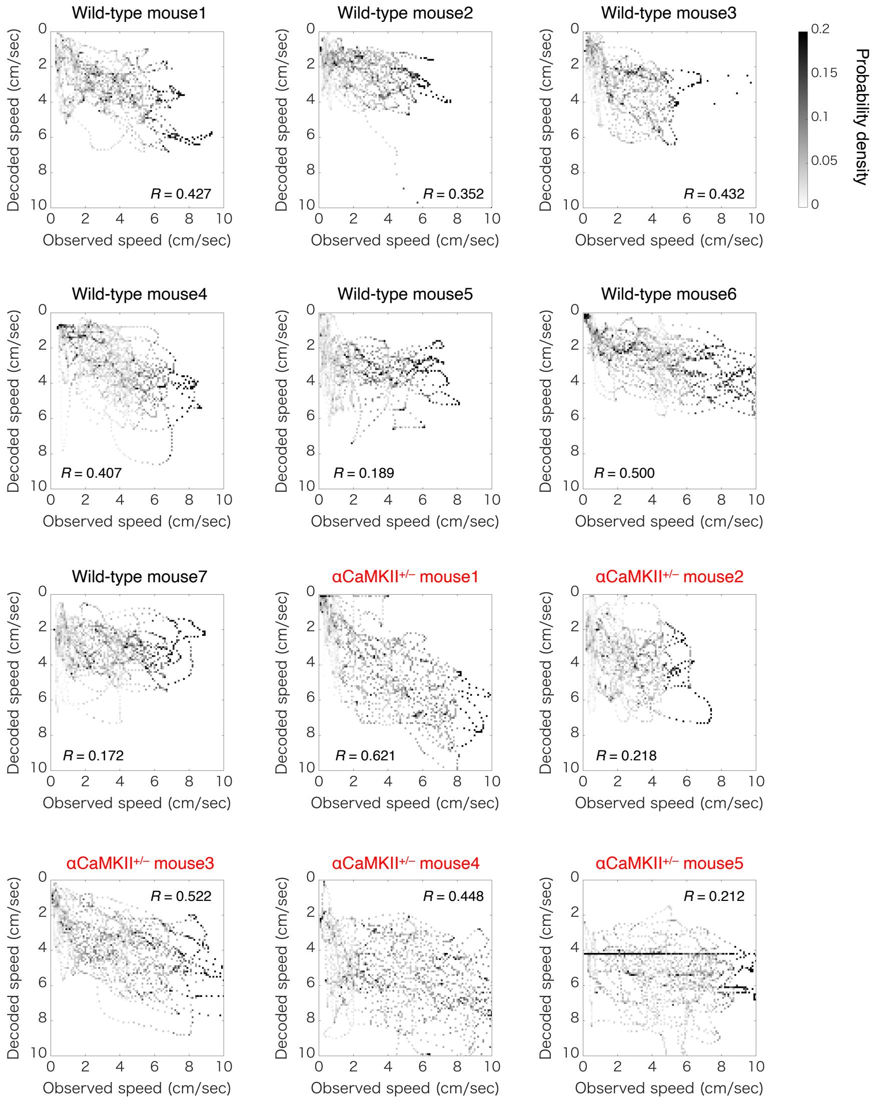

Figure S5. Speed decoding accuracy in the open field.

Confusion matrices for speed decoding results for all wild-type and αCaMKII$^{+/-}$ mice. The x-axis represents the actual mouse speed, and the y-axis represents the estimated mouse speed. Each column corresponds to the estimated probability distribution over speed for a given true speed (0.1 cm/sec bins). The correlation coefficient (*R*) between the observed and decoded speeds (same as in Figure 2e) is shown in each mouse.

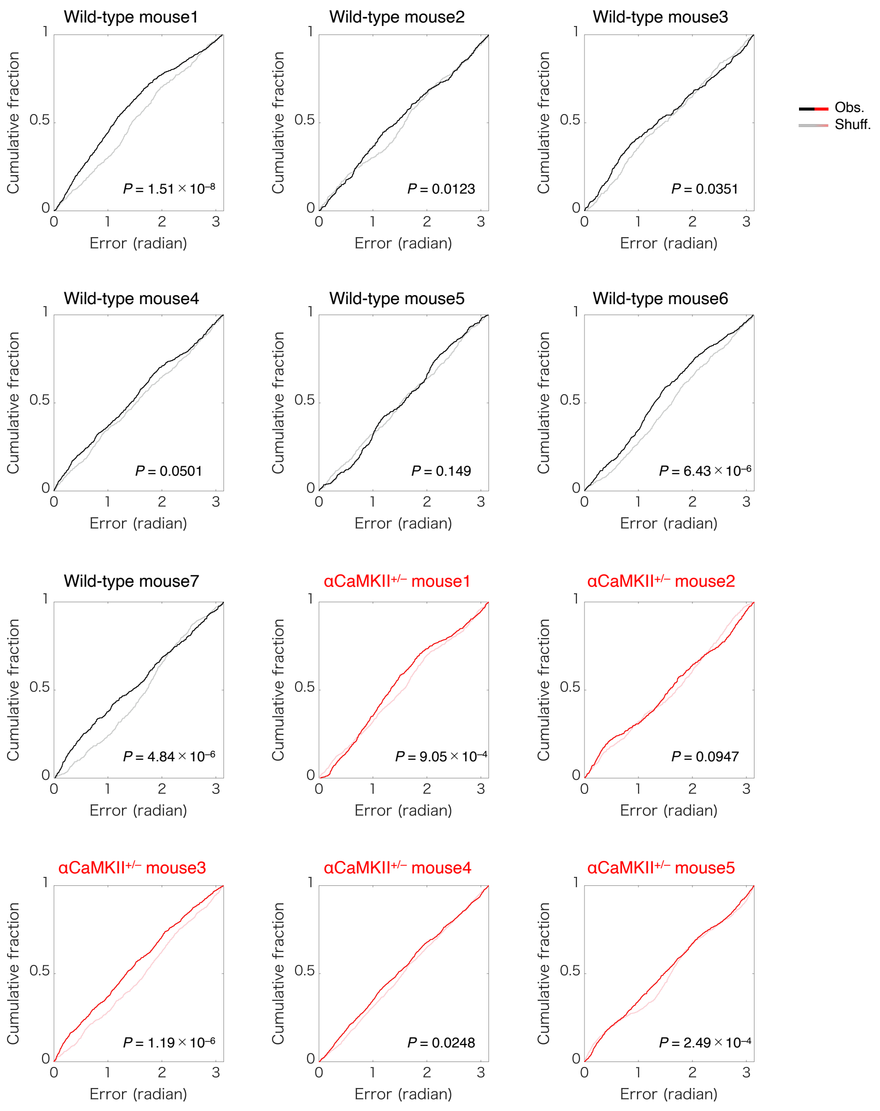

Figure S6. Direction decoding error in the open field.

Cumulative distribution functions (CDFs) of the direction decoding error. The results from all the mice (black; 7 wild-type mice, red; 5 αCaMKII$^{+/-}$ mice) are shown. Mean absolute errors (MAE, same as in Figure 2f) of each mouse are also shown in each figure. The *P*-value in each panel indicates the difference between the distribution of decoding error from observed and shuffled data (Kolmogorov-Smirnov test).

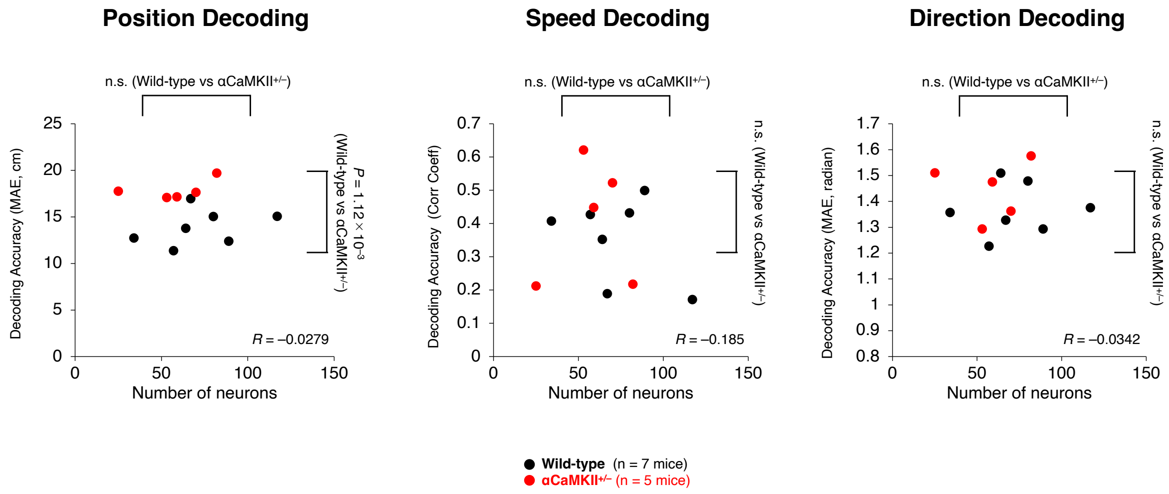

Figure S7. Decoding accuracies for position, speed, and motion direction are not sensitive to the numbers of neurons used for decoding.

Scatter plots showing the correlation between the decoding accuracies for position, speed, and motion direction and the number of the neurons used for decoding. Each black and red dot corresponds to individual wild-type and αCaMKII$^{+/-}$ mice, respectively. *R* values are correlation coefficients.

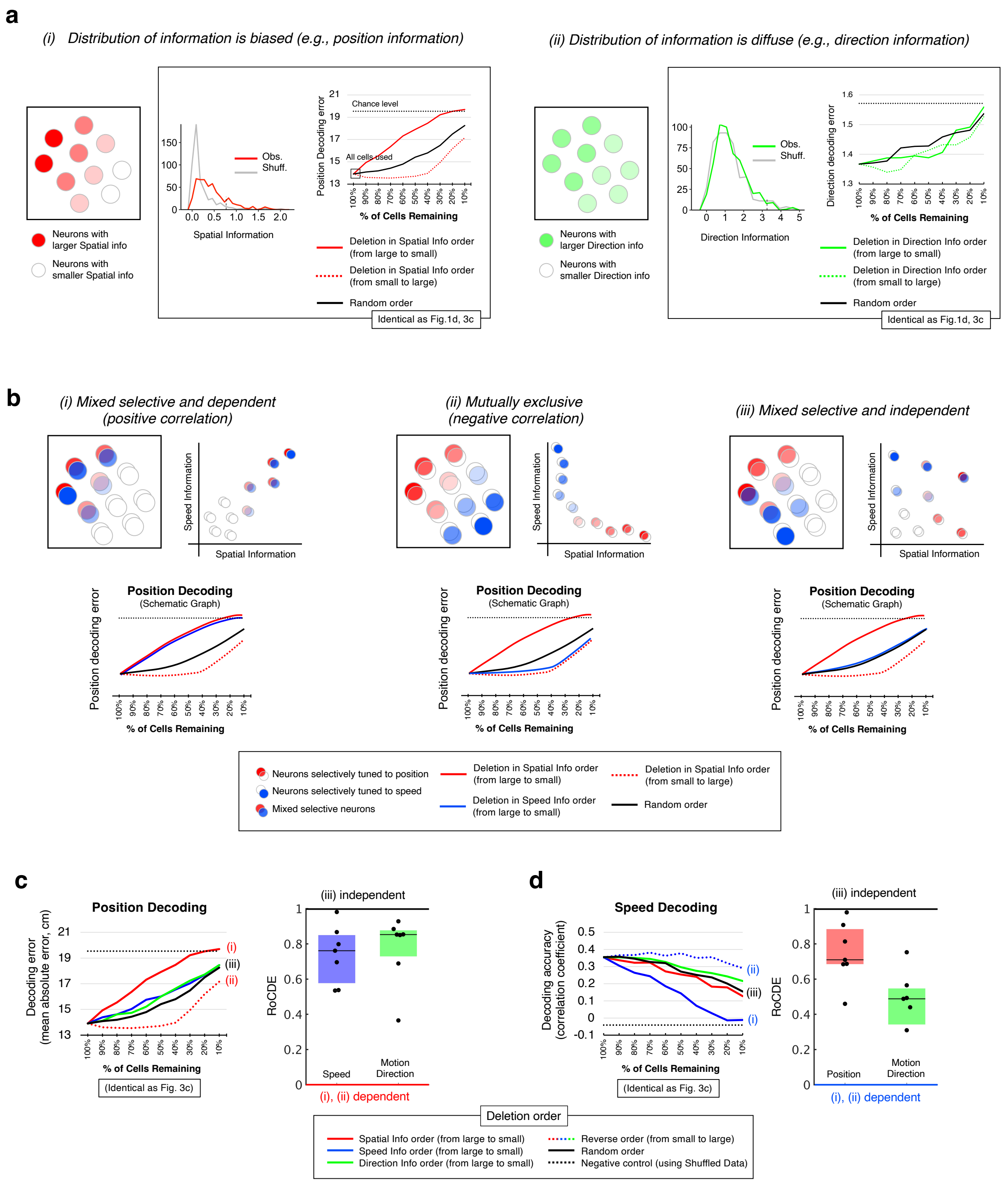

Figure S8. Quantitative evaluation of independency in the distribution of multiple types of information (in relation to Figure 3c).

a, The distribution pattern of one type of information. (i) distribution of information is biased to a specific population of neurons (e.g., position information): left, color density of the circles designates the amount of information; middle, distribution of information content (identical as Fig. 1d); right, changes in decoding performance when neurons are individually deleted (identical as Fig. 3c); In this case, when neurons are removed from datasets in the sequence of

spatial information from larger ones to smaller ones (solid red line in the right panel), the position decoding error increases faster than when cells are removed randomly (black line). When neurons are removed from the dataset in the sequence of spatial information from smaller to larger ones (dotted red line), the decoding error increases more slowly than when neurons are removed randomly. (ii) Information is diffusely distributed in the neuron population (e.g., direction information): the direction information of neurons is not significantly different from those of Shuff. (middle panel, identical to Fig.1d). In this case, when neurons are removed from datasets in the increasing or decreasing order of direction information (solid or dotted green line in the right panel), the decoding error does not increase faster or slower than when cells are removed randomly (black line). Information about motion direction is encoded by a population of dDG neurons, while individual dDG neurons do not apparently have a significant amount of directional information.

b, Three hypothetical results of analyses depending on three different models on the relationship between the distribution of two types of information (e.g., position and speed). (i) Mixed selective and dependent (positive correlation): Neurons have mixed or multiple selectivity, showing selective tuning to both position and speed. Also, neurons that encode large information about speed tend to encode large information about position simultaneously (red and blue circle, mixed selective), and the amount of encoded information is positively correlated with each other. In this model, if cells are removed in the order of speed information (blue line), the position decoding error would increase as in the case of removing cells in the order of spatial information (solid red line). (ii) Mutually exclusive (negative correlation): The population of cells that encode information about speed tends not to encode information about the position. In this model, if the neurons are deleted in the order of speed information (blue line), the accuracy of position decoding would be reduced in the same way as when the cells are deleted from those with smaller spatial information (dotted red line). (iii) Mixed selective and independent of each other: Information about position and speed encoded in a neuron is independent of each other. Some cells are mixed selective (encoding both types of information simultaneously), and the degree of coding for each variable is diverse. In this model, removing the cells from the datasets in the order of speed information (blue line) would reduce the accuracy of position decoding, as random order deletion does (black line).

c, The degree of the relative independency of the information distribution patterns between position information and others (identical as Figure 3c): To quantitatively measure the relative independency of the distribution between two information types, we calculated the ratio of

cumulative decoding errors (RoCDE) (for details, see methods section). The $RoCDE_{(Speed/Position)}$ and $RoCDE_{(Direction/Position)}$ are calculated for each individual wild-type mouse (right panel). The individual black dots correspond to individual wild-type mice. The center line, box boundaries for the boxplot indicate the median, upper and lower quartile of the data. The $RoCDE_{(Speed/Position)}$ and $RoCDE_{(Direction/Position)}$ are 0.739 ± 0.06 and 0.775 ± 0.07, respectively. They are close to 1 ((iii) independent, black line in right panel) rather than 0 ((i), (ii) dependent, red line in the right panel), suggesting that the distribution of speed and direction information is independent of that of position information.

d, The degree of difference of the information distribution patterns between speed information and others (identical as Figure 3c): The $RoCDE_{(Position/Speed)}$ is close to 1 (0.749 ± 0.06), suggesting that the distribution of position information is independent of that of speed information. $RoCDE_{(Direction/Speed)}$ is around 0.5 (0.425 ± 0.10), which indicates moderate dependency between direction and speed information. This is probably because speed-off cells tend to be unable to show fine-tuning for motion direction due to their low activity during motion, resulting in their mutually exclusive nature.

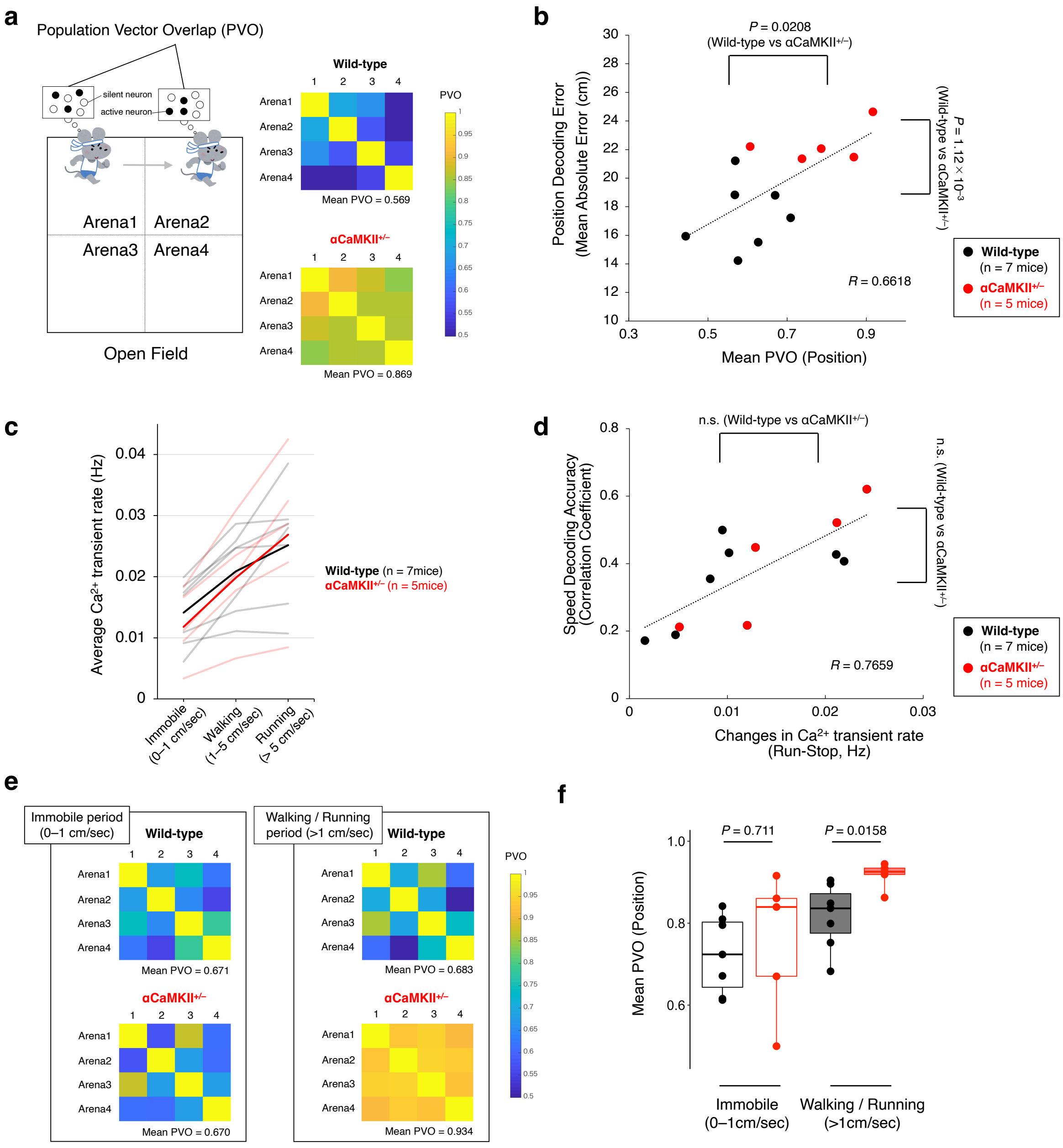

Figure S9. Position and speed decoding accuracies are correlated with the distinctness of neuronal activity patterns and activity frequency, respectively.

a, Similarities in the population activity patterns of dDG neurons among different areas in the open field. The open field is divided into 4 subareas (Arena 1–4), and the population activity patterns of dDG neurons are compared among these subareas. The similarities among the neural activity patterns are evaluated by the population vector overlap (PVO; for details, see Materials and Methods section) of the columns of the average Ca^{2+} transient rate across dDG neurons between two subareas. Color-coded PVO matrices and their mean PVOs are shown

for a representative mouse from the wild-type and αCaMKII$^{+/-}$ groups. A larger PVO indicates higher similarities in the neural activity patterns, and a smaller PVO shows that there are distinct neural activity patterns among subareas.

b, Scatter plot showing the correlation between the mean value of the PVO among all subareas and the position decoding accuracy for each mouse. Each black and red dot corresponds to an individual wild-type and αCaMKII$^{+/-}$ mouse, respectively.

c, Changes in the average Ca^{2+} transient rate with mouse acceleration. The vertical axis shows the average Ca^{2+} transient rates during the Immobile (<1 cm/sec), Walking (1–5 cm/sec), and Running (>5 cm/sec) periods. Each grey and pale red line indicates the average Ca^{2+} transient rates of an individual wild-type and αCaMKII$^{+/-}$ mouse, respectively. The black and red lines show the average of all mice in each group.

d, Scatter plot showing the correlation between the change in the Ca^{2+} transient rate (from the Immobile to Running periods) and speed decoding accuracy. Each black and red dot corresponds to an individual wild-type or αCaMKII$^{+/-}$ mouse, respectively.

e, Color-coded PVO matrices and their mean PVO in the Immobile period (0–1 cm/sec) and Walking / Running period (> 1 cm/sec) is shown for a representative mouse from the wild-type and αCaMKII$^{+/-}$ groups.

f, Box plot showing the mean PVO of wild-type and αCaMKII$^{+/-}$ mice in the Immobile and Walking / Running period. In the Immobile period, there was no significant difference in the mean PVO of wild-type and αCaMKII$^{+/-}$ mice ($P = 0.711$). On the other hand, the mean PVO of αCaMKII$^{+/-}$ mice was significantly larger than wild-type mice ($P = 0.0158$).

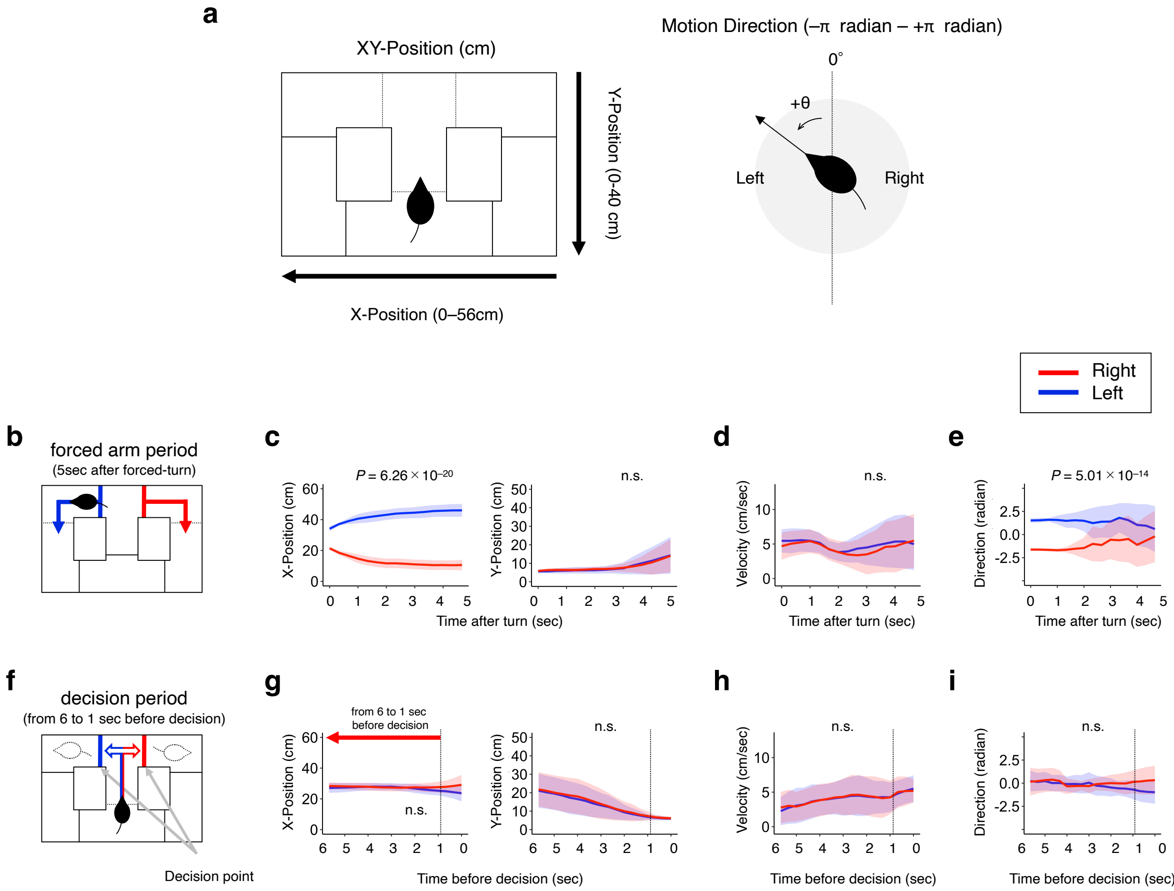

Figure S10. X and Y position, speed, and motion direction of mice during the forced arm and decision periods in the T-maze.

a, Definition of the X position (0–56 cm), Y position (0–40 cm) and motion direction (from –π to +π radians) for mice in the T-maze

b, The arm period is defined as the time period 5 sec after the forced turn.

c-e, Plots of the mean±SEM X and Y positions (c), speed (d), and motion direction (e) of the mice across all trials for the left (blue lines) and right (red lines) forced choices. The X position and motion direction in the arm period are significantly different between the left and right forced choices.

f, The decision period is defined as the time period from 6 to 1 sec before the decision.

g-i, Plots of the mean±SEM X and Y positions (g), speed (h), and motion direction (i) of the mice across all trials for the left (blue lines) or right (red) free choice. The X-position, Y

position, and motion direction in the decision period are not significantly different between the left and right choices.

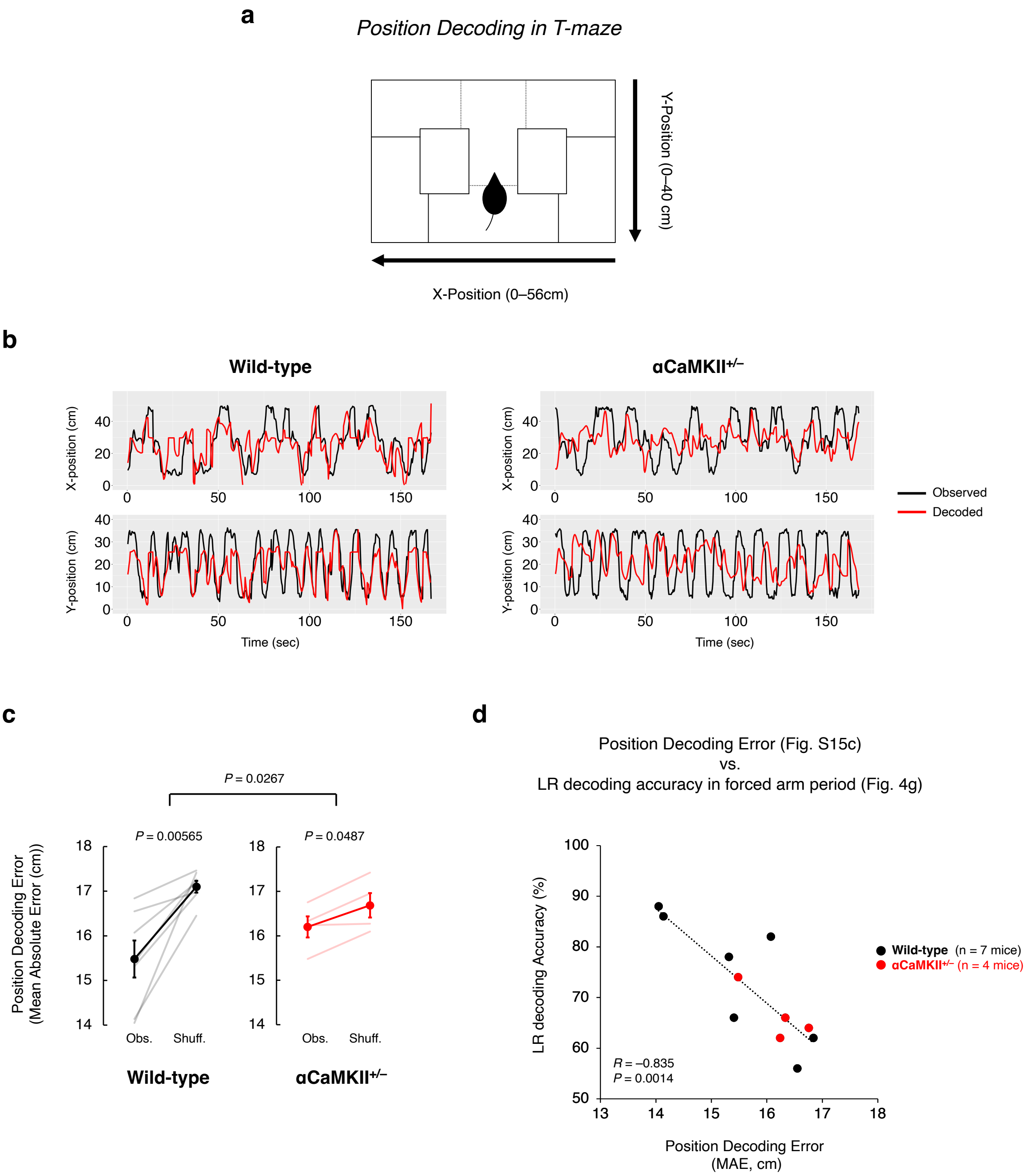

Figure S11. Position decoding in T-maze.

a, Decoding of the position information (x-y coordinates) in the T-maze. For decoding, the same machine learning method used for the open field (in Fig. 2) was used.

b, Representative results of position decoding in T-maze from wild-type mice (left panels) mice and αCaMKII$^{+/–}$ mice (right panels). The X and Y positions (cm) of mouse in the open field are shown in the upper and lower panels, respectively. The black lines show the observed X and Y positions of the mice (observed), and the red lines show the position decoded from the

population activity patterns of dDG neurons (decoded).

c, Accuracy of position decoding in 7 wild-type mice (left) and 4 αCaMKII$^{+/-}$ mice (right). The accuracy of the position decoding is reported as the mean absolute error. The black and red lines represent means, and the error bars indicate the standard error of the mean (SEM). The position decoding error of wild-type mice was significantly smaller than that of the shuffled data (paired *t*-test, $t(6)=4.205$, $P = 0.00565$). The decoding error of αCaMKII$^{+/-}$ mice was slightly smaller than the shuffled control (paired *t*-test, $t(3)=3.2176$, $P = 0.0487$) but significantly larger than wild-type mice (Obs.-Shuff. is compared; unpaired *t*-test, $t(10)=2.74$, $P = 0.0267$).

d, Scatter plot showing the correlation between the position decoding error (*SI Appendix*, Fig. S11c) and LR decoding accuracy in the forced arm period (Fig. 4g). There is a significant negative correlation ($R = -0.835$). The position decoding performance of αCaMKII$^{+/-}$ mice was less precise (MAE = 15.5–17 cm) than that of wild-type but LR decoding accuracy in the forced-arm period (average 65%) is significantly higher than the chance level (50%). Thus, even though the position decoding of x-y coordinates is not so precise (as in Fig. 2d and *SI Appendix*, Fig. S11c), the decoding performance of the current LR location can be better than the chance level. This may be because the defined area of the current LR location is much larger than the resolution of position decoding of x-y coordinates.

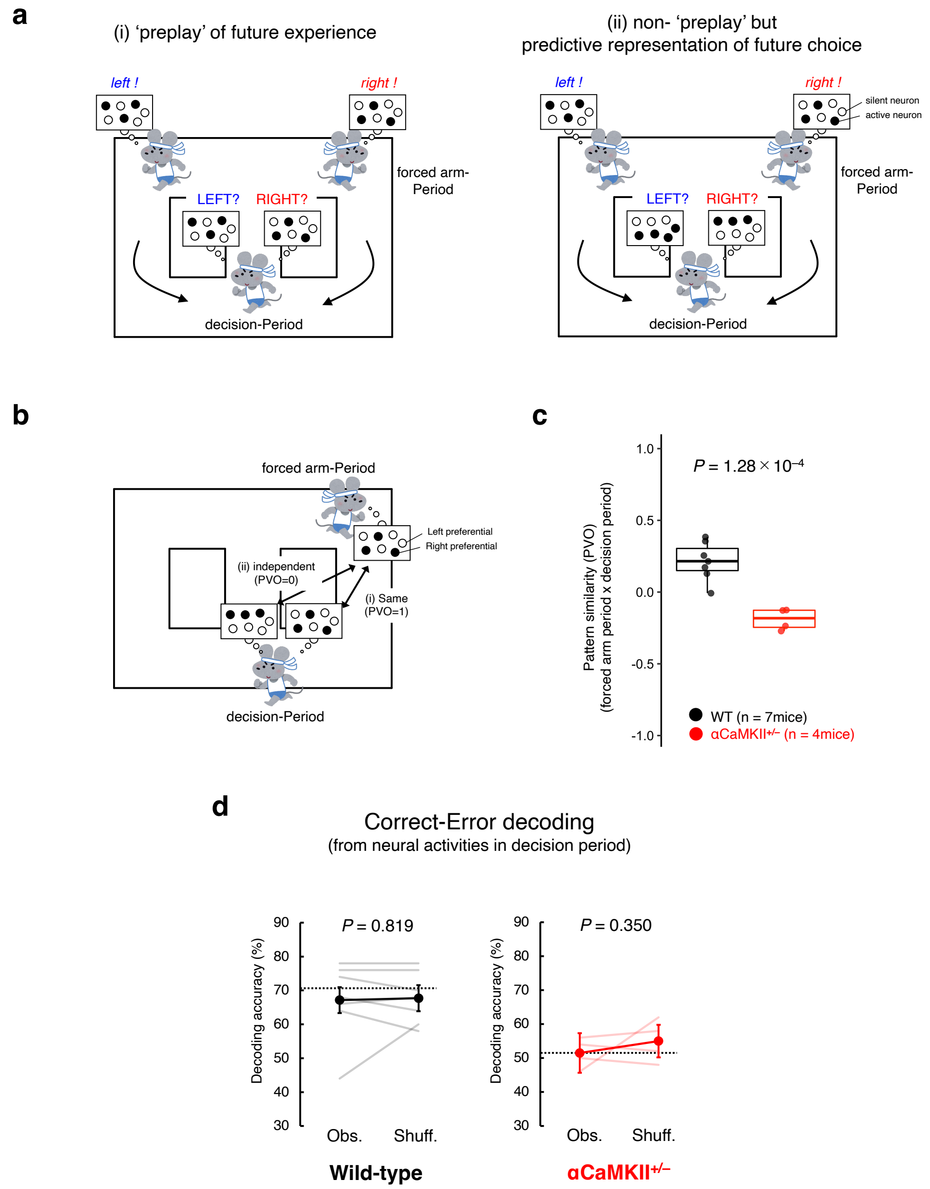

Figure S12. Relationship between the neural activities during forced arm and decision period in the T-maze test.

a, Two possibilities how the activities of DG neurons during the decision period represent the future LR location. (i) 'Preplay' of future experience: The neural activity representing the future LR location during the decision period is similar to those of the current LR location during the forced arm period. (ii) Non-'preplay' but predictive representation of future choice: The neural activities representing the LR of future and current location during the decision and forced arm period are different (in other words, independent or mutually exclusive).

b, Population vector overlap (PVO) indicating the similarity of the LR-tuning patterns of dDG neurons during the forced arm and decision period. If these patterns are more similar, the PVO would be closer to 1. If they are independent, it would be close to 0.

c, PVO of the LR-tuning patterns of neurons between the forced arm and decision period. An individual black or red dot corresponds to an individual wild-type or αCaMKII$^{+/-}$ mice, respectively. The PVOs of wild-type mice were significantly higher than those of αCaMKII$^{+/-}$ mice, but were relatively small (0.00 – 0.38).

d, Decoding accuracies on correct-or-error of choices in the forced alternation task. There were no significant differences in the decoding accuracies between Obs. and Shuff. either in wild-type or in αCaMKII$^{+/-}$ mice, indicating that correct or error cannot be decoded from activities in dDG neurons. Note that the decoding accuracy of Shuff. of wild-type mice is about 70% because the decoding accuracy expected by chance in correct-or-error is about 70%, due to the correct-error ratio of wild-type mouse is 71.4% (Fig. 4b).

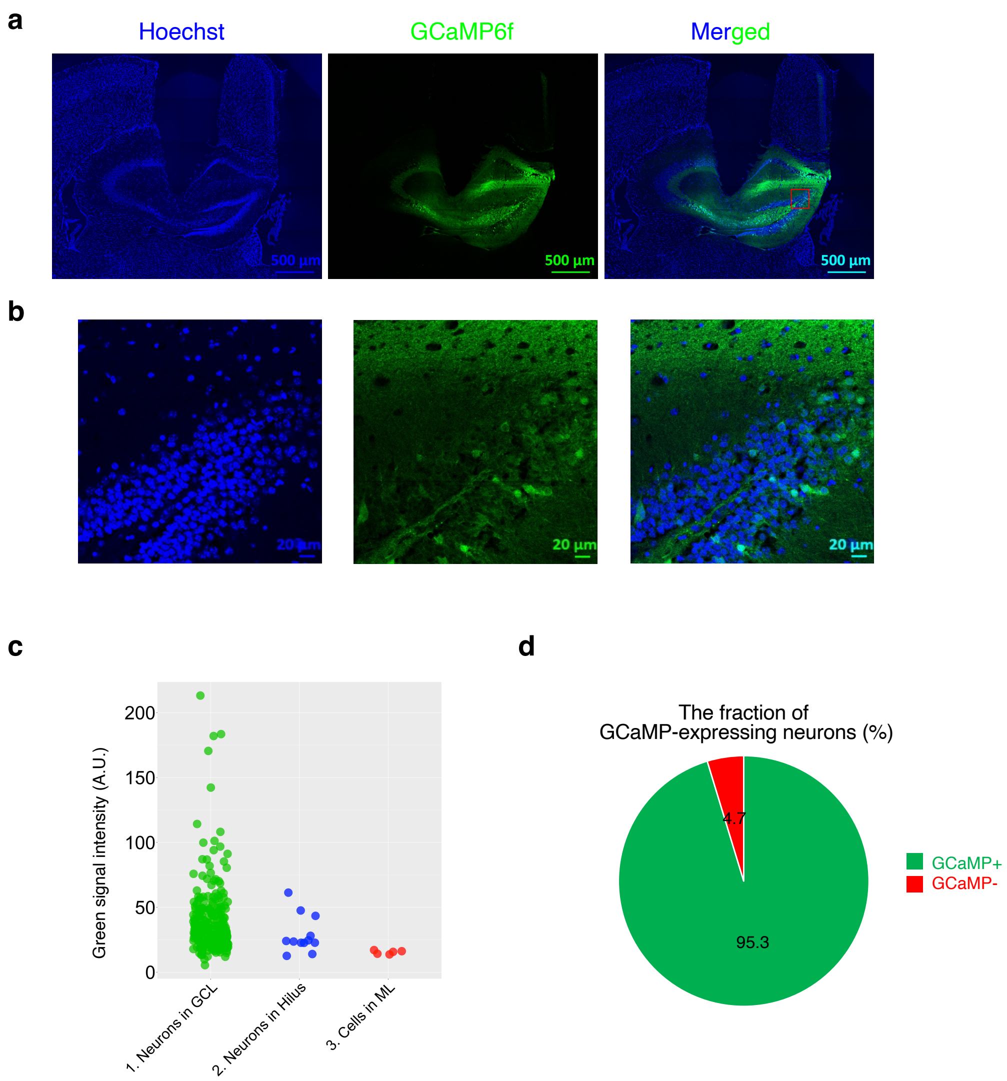

Figure S13. Placement of the GRIN lens implanted in the dentate gyrus.

a, Coronal slice of mouse brain showing the position of implanted GRIN lens. (left: Hoechst; middle: GCaMP6f; right: merged image).

b, Enlarged images of the area enclosed by the red rectangle in the panel (a).

c, The intensities of the GCaMP6f signal of the cell nucleus in the granule cell layer, hilus, and molecular layer.

d, The fraction of DG neurons expressing GCaMP6f out of all DAPI-positive cells in the granule cell layer and hilus.

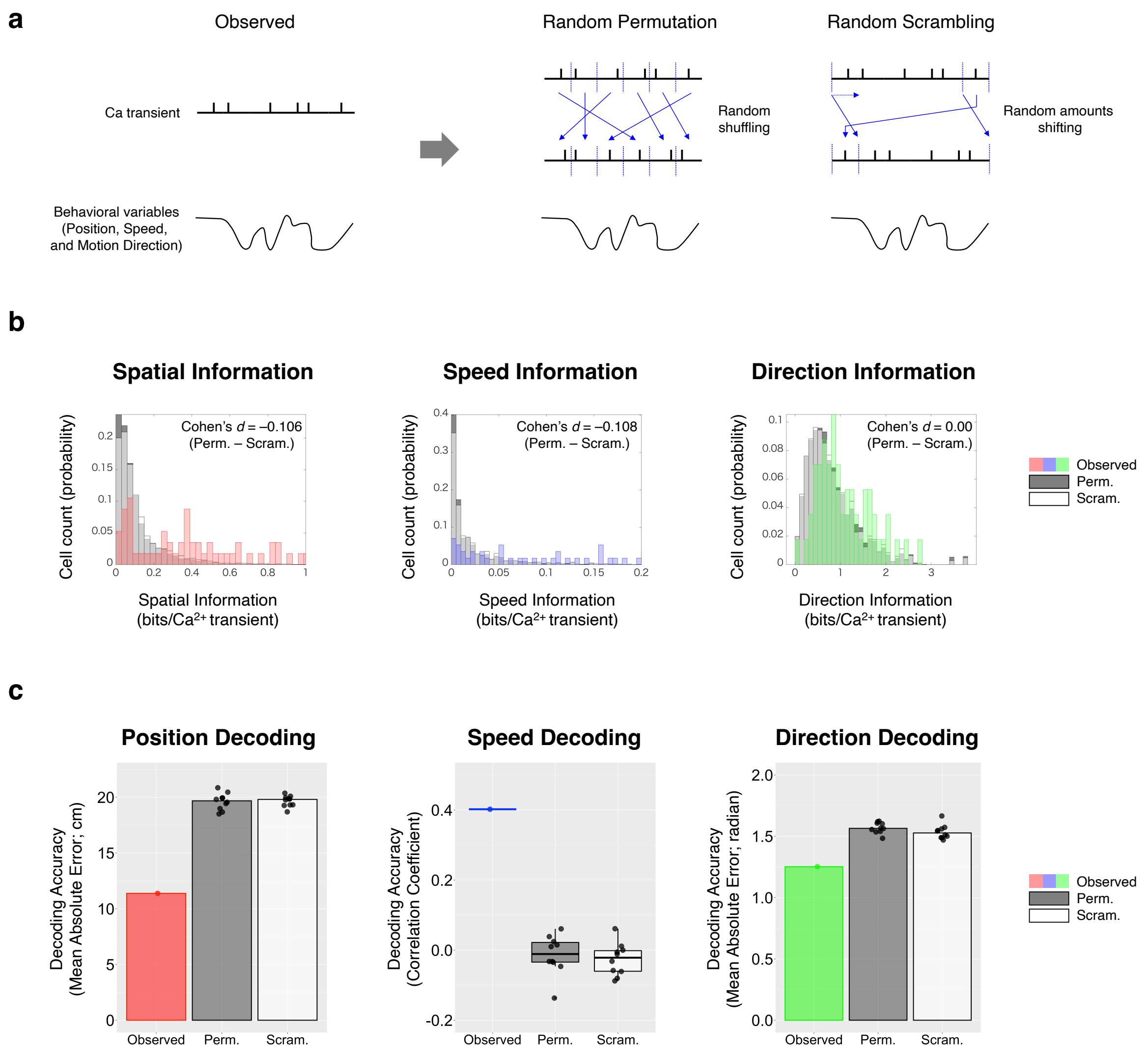

Figure S14. Different shuffling methods do not present significant differences regarding information statistics and decoding performance.

a, Different methods for generating shuffled data. The random permutation method divided the data into 1000 segments and randomly sorted them, resulting in shuffled data. Thus, both the correspondence between neural activity and behavioral data in the original data, as well as the dynamics of neural activity itself, is impaired. The random scrambling method shifts the time series of the neural activity by a random number of frames while maintaining the temporal dynamics of the neural activity. This method results in disrupted correspondence between the time series of neural activity and behavior; however, the temporal dynamics of behavior and neural activity is preserved.

b, Distribution of spatial, speed, and direction information from observed neurons ("Obs.",

color-coded bins), as well as for shuffled data (“Perm.”, dark gray) and scrambled data (“Scram.”, white), for a representative wild-type mouse. We repeated random permutation and random scrambling 1000 times each to generate shuffled data, and there was no significant difference between their distributions.

c, Decoding accuracy of position, speed, and motion direction by different shuffling methods. Random permutations and random scrambles were repeated 10 times each to generate shuffled data from a representative wild-type mouse. Decoding analysis was performed on these shuffled data, in which the accuracy was compared, and there was no significant difference between them.

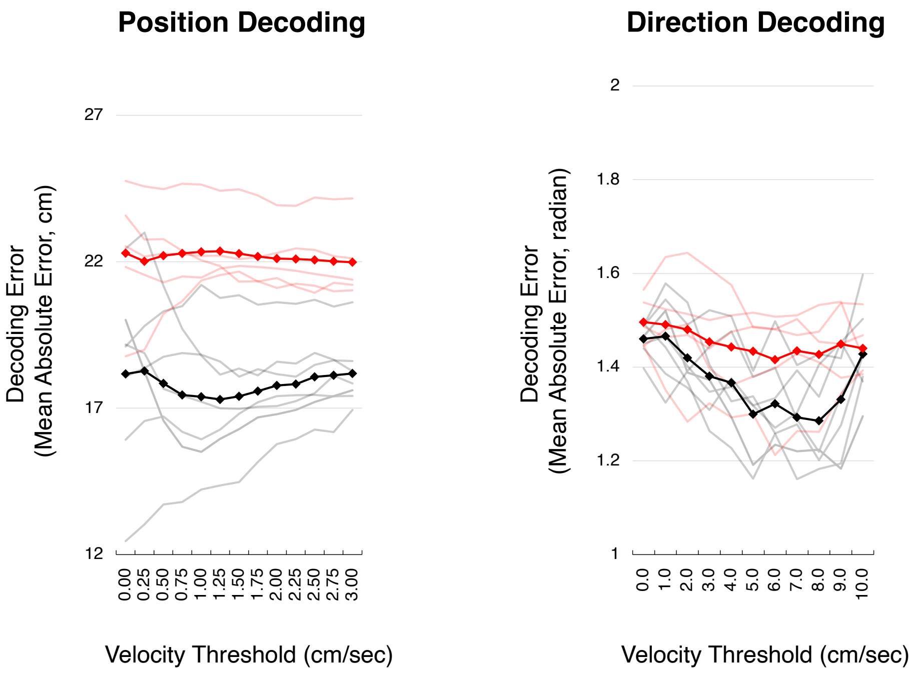

Figure S15. Velocity threshold and decoding accuracy for position and motion direction.

The horizontal axis indicates the threshold for velocity filtering (for details, see *SI Appendix, Supplementary Results* "Velocity filtering for position and direction decoding"). The vertical axis indicates the position decoding error (left panel) and direction decoding error (right panel). The grey and pale red lines indicate individual wild-type and αCaMKII$^{+/-}$ mice, respectively. The black and red lines show the average of all mice in each group.

Dataset S1.

Summary of information about the statistics used in the paper.

Supporting Information Text

Supplementary Results

Position and speed information is encoded in the dDG by a different coding principle (*SI Appendix*, Fig. S9).

We found the selective impairments of information (position in open field and future location in T-maze) in αCaMKII$^{+/-}$ mice (Fig. 2). There are two hypotheses on why a specific type of information is impaired in the DG of these mice.

(i) Different types of information are encoded by different cell populations (e.g., there are groups of place-coding cells and speed-coding cells in the DG which are separable, and one of them is selectively impaired).

(ii) Different types of information are encoded in the same or overlapping cell populations, but by different coding principles (e.g., in CA1 and EC, position information is represented in the activity patterns of neurons[1,2], and speed information is encoded by the frequency of the neural activity[3,4]), and one of them is selectively impaired.

The selective impairment in αCaMKII$^{+/-}$ mice is unlikely to be attributed to selective death or general functional impairment of specific groups of neurons because we found that neurons carrying position information also encode other types of information in wild-type mice. Instead, we assume that different types of information are encoded by different coding principles (e.g., in CA1 and EC, position information is represented in the activity patterns of neurons[1,2], and speed information is encoded by the frequency of the neural activity[3,4]) and that the coding principle for position information is selectively impaired in the dDG of αCaMKII$^{+/-}$ mice.

Then, at first, we examined the relationship between position decoding accuracy and distinctness of activity patterns of dDG neurons across the subareas in the open field. In our data, the population vector overlap (PVO) (see Materials and Methods section; *SI Appendix*, Fig. S9a), which measures how similar firing patterns are across different subareas in the open field, was significantly correlated with the error of position decoding (*SI Appendix*, Fig. S9b). Thus, the increased position decoding error in αCaMKII$^{+/-}$ mice relative to that in wild-type mice ($P = 1.12 \times 10^{-3}$, Fig. 2d) might be attributable to the larger mean PVO ($P = 0.0208$, *SI Appendix*, Fig. S9b), corresponding to less distinct firing patterns of neural ensembles across different subareas. Next, we investigated how encoding of speed information is associated with overall firing frequency of dDG neurons. We found positive and linear correlations between the speed of the mice and the average Ca^{2+} transient rate in the dDG (*SI Appendix*, Fig. S9c).

We also found a significant correlation between speed decoding accuracy and the change in the average Ca^{2+} transient rate with speed (*SI Appendix*, Fig. S9d), neither of which were significantly different between wild-type and αCaMKII$^{+/-}$ mice (Fig. 2e and *SI Appendix*, Fig. S 9d). Thus, information about position and speed might be encoded with different coding principles in the dDG, and only the coding principle for position may be affected in the dDG of αCaMKII$^{+/-}$ mice. In addition, we noted that the Ca^{2+} transient rate is increased with the increasing moving speed of mice (*SI Appendix*, Fig. S9c). We then divided all data into the Immobile period (0-1 cm/sec) and Walking / Running period (> 1 cm/sec) and measured the mean PVO of position in an open field (as in *SI Appendix*, Fig. S9a) in each period. In Immobile periods, the mean PVO of αCaMKII$^{+/-}$ mice was not significantly different from those of wild-type (*SI Appendix*, Fig. S9e and S9f). On the other hand, in the Walking / Running periods, the mean PVO of αCaMKII$^{+/-}$ mice was significantly higher than that of wild-type mice (*SI Appendix*, Fig. S9e and S9f). These results suggest that, in αCaMKII$^{+/-}$ mice, the population coding of position information is disorganized when the mice are running and the Ca^{2+} transient rate is high but not when the mice are immobile.

Previous studies have reported that increased neuronal excitability[5] and epileptiform activity[6,7] are observed in the DG and CA1 of αCaMKII knockout mice. It is also reported that hippocampal pyramidal neurons of mice with a point mutation in the αCaMKII show a deficit in LTP and stability of place cell[8,9]. Despite such increased excitability of neurons and the brain, in our data, the overall Ca^{2+} transient rate of dDG neurons in αCaMKII$^{+/-}$ mice was not significantly different from that of wild-type mice (*SI Appendix*, Fig. S9c). This may be because of the "synaptic homeostasis" that stabilizes the neuronal excitability and the neural firing rate, which was reported to be shared by multiple strains of autism spectrum disorder model mice[10]. It is also reported that these mice exhibit alternations in neural circuits that lead to abnormally high synchrony of network activity[11] and deficit in sensory information processing[12]. The authors discussed that imperfect homeostasis largely normalized firing rate but maladaptively compromised some aspects of the population coding, like firing synchrony and sensory tuning, in these mice[10]. It is tempting to speculate that, in αCaMKII$^{+/-}$ mice, that have dramatically reduced synaptic molecules (such as GluR1, GluR2 and PSD95), synaptic homeostasis could have increased neuronal excitability to stabilize overall firing rate but compromised the coordinated network activity of neurons and population coding of position information. On the other hand, speed information in the dDG of αCaMKII$^{+/-}$ mice was not disturbed, possibly because it depends on changes in the overall neural firing frequency, which may not affected by compromised coordinated network activity. Thus, the dDG may utilize different coding principles for encoding different types of information, which supports the notion that individual

neurons can independently participate in encoding different types of information.

Neural representation of past and future states in T-maze by populations of dDG neurons (*SI Appendix*, Fig. S12).

We investigated the relationship between the neural representations of the current location in the forced arm period and those of the future state in the decision period. There are at least two possibilities for these relationships (*SI Appendix*, Fig. S12a). One is that the representations of information about left and right in the decision and forced arm period are similar to each other, and neural activity in the decision period preplays the predicted future experience in the next forced arm period ((i) in *SI Appendix*, Fig. S12a). Another is that the representation of future LR information in the decision period is different from those in the forced arm period ((ii) in *SI Appendix*, Fig. S12a).

In Fig. 5a, we noted a small but significant correlation between the LR indices of the forced arm and decision periods in wild-type mice ($R = 0.2281$, $P = 7.91 \times 10^{-5}$; Fig. 5a). A small number of neurons in wild-type mice shows similar activities during the forced arm and decision period (e.g., cells in the upper right or bottom left corners in Fig. 5a show preferential activity for right or left, respectively), suggesting that these neurons might be involved in ‘preplay’-like activities. Then, we sought to examine whether there was an overall association between the LR-tuning patterns of neurons in the forced arm and decision periods. First, we calculated the PVO (see Materials and Methods section) of the column vector of the LR index for all cells between the forced arm and decision periods for each mouse. When these two vectors are similar to each other, PVO would be close to 1, and when they are independent of each other, PVO would be close to 0 (*SI Appendix*, Fig. S12b). The PVOs of wild-type mice were significantly higher than those of αCaMKII$^{+/-}$ mice, but were relatively small (0.00 – 0.38, *SI Appendix*, Fig. S12c). Overall LR-tuning patterns in the forced arm and decision period were slightly but significantly similar to each other. These results suggest that a small number of neurons might be involved in ‘preplay’-like activity during the decision period ((i) in *SI Appendix*, Fig. S12a), while the majority of neurons may not.

Next, we investigated the associations between the importance of neurons in the population coding during the forced arm and decision period. In Fig. 5c, removing neurons that are important for LR decoding in the forced arm period did not have a significant impact on those in the decision period and vice versa. This result indicates that the neuron populations used for the decoding of left and right in the forced arm and decision period are not similar to each other but rather independent. Therefore, the major representation of future LR information in the decision period that was used for decoding is not likely to be ‘preplay’-like activities of

future experience similar to that in the forced arm period ((i) in *SI Appendix*, Fig. S12a) but rather predictive representations of the future state that are independent of the representations during the force arm period ((ii) in *SI Appendix*, Fig. S12a).

The traditional view of place coding in the hippocampal circuit is that the place cells encode the current location of the animal. It has also been known that neural activity patterns in CA1[13,14], CA3[14,15], and medial EC[16] are involved in the prospective representation of future states. In our analysis, we were able to decode whether the mice were on the left or right side of the T-maze by the population activity patterns of dDG neurons either after or before a left or right turn. These results suggest that the population activity patterns of dDG neurons not only encode the current location but also may be involved in the predictive representation of future states alongside other hippocampal regions. These two states are represented in the dDG in a partially similar but largely independent manner.

Velocity filtering for position and direction decoding (*SI Appendix*, Fig. S15).

The activation of hippocampal place cells during immobile periods, which occurs in conjunction with hippocampal sharp wave ripples (SWRs), is known not to represent the current position of animals[17,18]. Therefore, in analyses of position information in the hippocampus, periods of immobility are generally excluded from the data. We did not know whether immobile periods should also be excluded for data obtained from the dDG, as it is not known whether neural activity equivalent to SWRs can be observed in the Ca^{2+} imaging in this region. Therefore, to test the validity of this procedure, we examined whether removing periods of immobility would improve the accuracy of position decoding in the dDG (*SI Appendix*, Fig. S15, left panel). We removed the time bins in which the movement speed of the mouse was below the threshold (from 0.0 cm/sec to 3.0 cm/sec) and performed position decoding using the remaining data. In wild-type mice, removing time bins in which the speed was below 1.0–1.25 cm/sec reduced the position decoding error (*SI Appendix*, Fig. S15, left panel). These results suggest that neural activity during periods of immobility that do not represent current location may also be observed in the Ca^{2+} imaging data of the dDG. Based on these results, all periods in which the mouse's speed was below 1 cm/sec were eliminated from the analysis of position information. (This threshold is roughly equivalent to those commonly used in hippocampal CA cells (1.0–2.0 cm/sec)[19]).

The motion direction of the mouse was estimated from the changes in the mouse position in the open field. Even small movements such as grooming, rearing or head turning were calculated as actual mouse movements. To exclude these periods and more accurately measure motion direction, we examined the speed threshold below which movements could be

removed from the datasets (*SI Appendix*, Fig. S15, right panel). We removed all the time bins in which the speed of the mouse was below the threshold (from 0.0 cm/sec to 10.0 cm/sec) and performed direction decoding using the remaining data. In wild-type mice, the decoding error for motion direction decreased after applying a threshold of 4.0–8.0 cm/sec to remove movement periods, suggesting that small movement periods below these thresholds were useless for accurate direction decoding (*SI Appendix*, Fig. S15, right panel). Based on these results, all periods when the mouse ran slower than 4.0 cm/sec were removed from our datasets for the analysis of motion direction.

On the possibility that information about motion direction is decoded from place-coding cells.

There might be a possibility that motion direction was decoded from the activities of the neurons that encode spatial information, not from those encoding information about motion direction. If the decoding of motion direction was achieved by an activities of neurons encoding spatial information through an association between position and motion direction, deleting neurons in the order of spatial information (red line in Fig.3c right panel) would increase decoding errors faster than that of random order deletion (black line). However, in Fig. 3 and *SI Appendix*, Fig. S8, deleting neurons in the order of spatial information (red line) did not affect the accuracy of direction decoding faster than random order deletion (black line) did. These results indicate that neurons with relatively larger spatial tuning were not preferentially used for motion direction decoding. Therefore, the decoding accuracy of motion direction is not likely to be achieved by an activity of the neurons encoding position information.

Also, our results of decoding analysis of αCaMKII$^{+/-}$ mice supported the idea that our decoding of position and motion direction are independent. In αCaMKII$^{+/-}$ mice, position information was selectively impaired, but direction information was not significantly different from wild-type mice (Fig.2d and 2f). This result also suggests that successful decoding of direction information is not achieved by an association between position and direction.

Supplementary Methods

Estimation of the active population of DG neurons.

Estimation of the active population of DG neurons was performed on the basis of the instructions of Dr. Jonathan Zapata, Inscopix Inc.. In brief, after motion correction of the raw fluorescent images from the maximum intensity projection of GCaMP fluorescence images, the total number of neurons in a field of view was manually counted. Likewise, the maximum intensity projection of the fluorescence change ratio ($\Delta F/F$) images was generated, and the total number of active neurons during the 30 min recording was manually counted. The maximum projections of GCaMP fluorescence images and $\Delta F/F$ images were calculated throughout all frames in the 30 min recording in the OF using the "movie projection" tool available in IDPS and saved as tiff files. We counted the number of cells in the maximum projection image frames using the multipoint tool of ImageJ (available from https://imagej.nih.gov/ij/). To confirm the activity of all the detected cells, we manually drew regions of interest (ROIs) over all the cells in the $\Delta F/F$ projection images and confirmed the traces of the Ca^{2+} signals with IDPS. All neurons that exhibited calcium transients individually in the soma at least once during the entire recording period were considered "active". All the image data that were used to perform active cell counting and the detailed method are available at "SSBD: repository" database [http://ssbd.qbic.riken.jp/set/20200603/].

Behavioral experiments.

Before every behavioral experiment, the OF or T-maze apparatus was cleaned using weakly acidified hypochlorous water (super hypochlorous water; Shimizu Laboratory Supplies, Kyoto, Japan) to prevent bias due to olfactory cues. All behavioral experiments were carried out in a sound-proof room, and the behavior of the mice was monitored through a computer screen located outside the room to minimize artefactual cues due to the presence of the experimenter. Mouse behavior was recorded at a 3 Hz sampling rate. For the OF and T-maze, different groups of mice were used (OF, 7 wild-type mice and 5 αCaMKII$^{+/-}$ mice; T-maze, 7 wild-type mice and 4 αCaMKII$^{+/-}$ mice).

Open field test

More than a week after baseplate attachment, mice were habituated to the test environment. Each mouse was lightly anaesthetized, and a dummy camera (Inscopix, CA) was mounted on the mouse. At least 30 min after recovery from anesthesia, mice were placed for 2 hr in the OF

arena (40 cm × 40 cm × 30 cm; width, depth, and height, respectively; O'Hara, Japan), made of opaque white plastic. The OF apparatus was evenly illuminated with 100 lux white LED light installed above the apparatus. This habituation session was repeated for three days. One day after the final habituation session, OF experiment was performed. Prior to the experiment, the mice were weakly anaesthetized with isoflurane, and an nVista miniature microscope was mounted onto the head stage. The mice were then habituated in the testing room for at least 30 min after recovery from the anaesthesia. Following the habituation in the testing room, each mouse was placed in the OF arena, and neuronal activity was recorded for 30 min. In order to obtain the location time sequence of each mouse, the images of the mouse were automatically processed by an ImageJ plugin (Image OF, freely available on the Mouse Phenotype Database website: http://www.mouse-phenotype.org/software.html).

T-maze test

The T-maze test was conducted using an automatic T-maze apparatus (O'Hara, Japan) as previously described[20]. In brief, the maze consists of the stem of the T (13 cm × 24 cm), the left and right (L/R) arms (11.5 cm × 20.5 cm each side), and connecting passageways from the end of the L/R arms to the starting compartment. These compartments are partitioned by sliding doors that open downward. The mice were subjected to a spontaneous alternation protocol for five sessions, with at least one day (two days maximum) of rest between sessions. Each session consisted of 10 trials with a 50-min cut-off time, and each trial consisted of a first and second run. On the first run, the mouse was forced to choose one of the L/R arms (forced choice). After the mouse was in the L/R arm for more than 10 sec, the door to the connecting passageway was opened, which allowed the mouse to return to the starting compartment. When the mouse returned to the starting compartment, all the doors of this component were closed; then, after 3 sec, the doors connecting to the L/R arms opened for the second run, in which the mouse could freely choose either of the L/R arms (free choice). The percentage of trials in which the mouse entered the arm opposite to their forced-choice run (the "correct" arm) was calculated. The choice of the L/R arm for the forced trials was varied pseudo-randomly across trials using a Gellermann schedule so that the mice received equal numbers of left and right presentations. Data acquisition, control of the sliding doors, and data analysis were performed with ImageTM software (freely available on the Mouse Phenotype Database website: http://www.mouse-phenotype.org/software.html)

Definitions of the position, speed, and motion direction of mice.

(i) Position: The OF arena (which had an area of 40 cm × 40 cm) was represented as 200 × 200 pixel grid. The position of the mouse is determined from the centroid of its shadow on the camera. We then assigned a label corresponding to the discrete location of the mouse (e.g., [10, 100]) to each time bin (=1/3 sec).

(ii) Speed: From the distance travelled between 1 sec before and after a given time point, we calculated the speed of the mouse at that moment and assigned this speed (cm/sec) to each time bin.

(iii) Motion direction: The visual tracking system that we used does not allow direct measurement of head direction. Instead, we indirectly estimated the direction of motion from the changes in the position of the mouse. The motion direction (in radians) was computed from the direction of change in two subsequent mouse positions (1 sec before and after a given time point) in the x-y plane and assigned this to each time bin. North was defined as 0 radians; west was defined from 0 to π radians, and east was defined from 0 to –π radians.

Statistical analysis of spatial, speed, and direction information.

To quantify the tuning specificities of neurons with position, speed, and motion direction, we measured their specificity in terms of the information rate of cell activity. We defined them as (i) spatial, (ii) speed, and (iii) direction information[21]. The Ca^{2+} event rate in Ca^{2+} imaging is considerably lower than in electrophysiological recordings (on average, approximately 30 Ca^{2+} transients per 30 min session). If the discretization of position, speed, and motion direction is too fine for the number of events in the recorded cells, we will not be able to obtain a proper null distribution when creating the shuffle data for that cell. Therefore, we set the resolution of the discretization of position and speed to be lower than those commonly performed.

(i) We used a 2×2 square grid to measure spatial information and computed the amount of Shannon information that a single Ca^{2+} transient conveyed about the animal's position. The spatial information I (bits per Ca^{2+} transient) of a cell was calculated as the mutual information score between the occurrence of a single Ca^{2+} transient of the cell and the animal's behavioral state of position using the formula:

$$I_{(\mathrm{bits/Ca^{2+}\ transient})} = \sum_{i=1}^{N} p_i \frac{r_i}{r} \log_2 \frac{r_i}{r}$$

where i is the bin number corresponding to the physical parameter (in this case, spatial position in the OF; i = 1–4 from the 2×2 square grid), N is the total number of bins, p is the probability that the mouse occupied bin i, r_i is the mean transient rate at bin i, and r is the overall mean

transient rate.

(ii) To measure speed information, we applied the same formula to the speed after discretizing it to a binary state: Run (for speeds >1 cm/sec) and Stop (for speeds <1 cm/sec).

(iii) Similarly, we measured direction information by applying the same formula to the motion direction after discretizing the full angle to 8 bins of 45 degrees each.

LR indices of neurons in the T-maze test.

To quantify the left- or right- preference of each neuron's activity for the current and future location in the T-maze, we generated a parameter called the LR index. For each neuron, we measured the average Ca^{2+} transient rates during the forced arm and decision periods for the left- and right-choice trials—that is, when the mouse is in the left or right arm of the T-maze, respectively—and computed the LR index as follows:

$$\text{LR index } = \log_2\left(\frac{R_R}{R_L}\right)$$

where R_L is the average Ca^{2+} transient rate across all left-choice trials, and R_R is from the right-choice trials. All neurons are assigned an LR index in both the forced arm and decision periods. A positive value indicates a neuron's preference for the left arm, whereas a negative value indicates a neuron's preference for the right arm.

Data Shuffling.

To assess the statistical significance of information coding of individual neurons, we computed chance distributions of the shuffled data using two common methods previously described in the literature[19,3,22].

Random permutation permutates calcium events (*SI Appendix*, Fig. S14). We divided the calcium event data into 1000 segments along the time axis and randomly sorted them to generate permutated data of calcium events. This method destroys temporal structures of neural activity and temporal correlations between neural activity and behavioral variables (e.g., position, speed, and motion direction in open field test); however, the overall neural activity is maintained across cells. We repeated this procedure 1000 times to obtain the distribution of 1000 shuffled data. For single-cell statistics, we compared the original information of individual cells with the null distributions of 1000 values of shuffled data generated from the original cell (*SI Appendix*, Fig. S2). If the original value of information of a cell exceeded 3 sigmas from the shuffled distribution, the cells were defined as carrying significant amounts of

information. For group comparison (for example, Obs. vs Shuff. in Fig. 1d, 4c, and 4d), we pooled all the shuffled data in a group together, and distributions of the original cells were compared with null distributions of the shuffled data.
Random scrambling is a method that maintains temporal dynamics of neural activity data while disrupting the relationship with behavioral patterns (for example, the calcium event timeseries and the animal's position (*SI Appendix*, Fig. S14)). We shift the whole vector of the calcium event time series in time by a random amount in a torus; that is, points beyond the data's time limits were reinsert from the other side. This procedure disrupts the relationship between neural activity and animal behavior, but preserves the temporal patterns of these variables. By repeating this procedure while changing the number of frames to be shifted at random, we obtain the null distributions of shuffled data (*SI Appendix*, Fig. S14). Information statistics are performed in the same way as the first shuffling method.
Since we found that the information statistics and decoding results of the shuffled data did not differ significantly between these shuffling strategies (*SI Appendix*, Fig. S14), we adopted the random permutation method for the generation of shuffled data.

Decoding position, speed, and motion direction in the open field.
To determine how the OF behavioral parameters are encoded in the DG, we trained decoders with machine learning methods to separately predict position, speed, and motion direction from the population Ca^{2+} activity. We assigned the labels of the discretized behavioral parameters of the mouse (position (cm), speed (cm/sec), and motion direction ($-\pi$ – $+\pi$ radians)) and the binary values of the Ca^{2+} signal (0 or 1) of all neurons to each time bin. We then divided the Ca^{2+} imaging data and behavioral data from each 30 min trial into the first 15 min and last 15 min halves, which were designated training and test data, respectively. For each pair of behavioral parameters (position, speed, or motion direction) and the value of the Ca^{2+} signal in the training data, we trained the decoders with one of eight different machine learning methods (Dense Feedforward Neural Network (DNN), Gated Recurrent Unit (GRU), Long Short-Term Memory (LSTM) network, Recurrent Neural Network (RNN), Support Vector Regression (SVR), Wiener Cascade (WC), Wiener Filter (WF), Extreme Gradient Boosting (XGB); the codes were obtained and modified from Glaser *et al*., eNeuro, 2020[23]). The decoding accuracy of the position/motion direction is reported as the mean absolute error in the distance between the predicted and actual position/motion direction. In the case of speed, decoding accuracy was reported as the correlation between the predicted and actual instantaneous speed. This is because speed, unlike position and motion direction, is not limited to a certain range, and the

mean absolute error of speed may depend on the average locomotion speed of each individual mouse, which would be inappropriate for the evaluation of decoding error. To assess the statistical significance of the decoding accuracies, the decoding error from the observed data was compared with that of the shuffled data, which is created by dividing the Ca^{2+} imaging data into 1,000 segments and sorting them randomly (see also *SI Appendix*, Fig. S14). Furthermore, we compared the decoding accuracies of the three behavioral parameters among the 8 decoders and found that they were not significantly different (*SI Appendix*, Fig. S3). Consequently, we reported the decoding results obtained with the LSTM Network, which showed slightly better decoding performance in wild-type mice than others. For decoding position and motion direction, we identified and removed time bins when the mouse moved at speeds below 1.0 cm/sec and 4.0 cm/sec, respectively, to obtain optimal decoding results. Details on the methods used in thresholding the data according to the movement speeds of the mice are described in the *SI Appendix, Supplementary Results* “Velocity filtering for position and direction decoding” and *SI Appendix*, Fig. S15.

Decoding current and future location in the T-maze test.

We similarly sought to decode the left or right preferences for the current and future locations in the T-Maze test using the population Ca^{2+} activity with machine learning methods. The decoders used for current and future LR locations are independently trained using neural activities during the forced arm and decision period, respectively. For each trial of the forced arm and decision periods, we assigned a label corresponding to the left or right choice of the mouse and the average Ca^{2+} transient rate of each neuron during the period. We then used a support vector machine (SVM) classifier function in MATLAB for binary classification of the left or right decision (MathWorks, MA). All 50 trials from each mouse were used for 25-fold cross-validation; the 50 trials were randomly divided into 48 trials of training data and 2 trials of test data. We repeatedly trained the binary classifier using randomly selected training data and performed left or right predictions for the remaining test data to evaluate the decoding accuracy. We also shuffled the Ca^{2+} transient data and performed the same decoding analysis. As with the open field behavioral parameters, the decoding accuracy was compared between the shuffled and unshuffled data.

Population vector overlap (PVO).

To quantify the similarities in population activity patterns of neurons between different

locations in the DG, we calculated the population vector overlap (PVO)[24]. The PVO for a population of N neurons in two different conditions (x, y) was defined as

$$PVO(x,y) = \frac{\sum_{i=1}^{N} \lambda_i(x)\lambda_i(y)}{\sqrt{\sum_{i=1}^{N} (\lambda_i(x))^2}\sqrt{\sum_{i=1}^{N} (\lambda_i(y))^2}}$$

where *i* is the neuron number; $\lambda_j(x)$ and $\lambda_j(y)$ are the average Ca^{2+} transient rates of neuron j in conditions x and y, respectively; and *N* is the total number of neurons. For a given population of neurons and a pair of conditions, a lower PVO indicates that the activity patterns of the neurons are distinct between the two conditions.

The ratio of cumulative decoding errors (RoCDE).

RoCDE between speed/direction information and position information and those between position/direction information and speed information are defined as blow:

$$\mathrm{RoCDE}_{(\mathrm{Speed/Position})} = \begin{cases} \dfrac{\mathrm{AUC_{Speed}} - \mathrm{AUC_{Random}}}{\mathrm{AUC_{Spatial}} - \mathrm{AUC_{Random}}} & (\mathrm{AUC_{Speed}} \geqq \mathrm{AUC_{Random}}) \\ \dfrac{\mathrm{AUC_{Speed}} - \mathrm{AUC_{Random}}}{\mathrm{AUC_{RevSpatial}} - \mathrm{AUC_{Random}}} & (\mathrm{AUC_{Speed}} < \mathrm{AUC_{Random}}) \end{cases}$$

$$\mathrm{RoCDE}_{(\mathrm{Direction/Position})} = \begin{cases} \dfrac{\mathrm{AUC_{Direction}} - \mathrm{AUC_{Random}}}{\mathrm{AUC_{Spatial}} - \mathrm{AUC_{Random}}} & (\mathrm{AUC_{Direction}} \geqq \mathrm{AUC_{Random}}) \\ \dfrac{\mathrm{AUC_{Direction}} - \mathrm{AUC_{Random}}}{\mathrm{AUC_{RevSpatial}} - \mathrm{AUC_{Random}}} & (\mathrm{AUC_{Direction}} < \mathrm{AUC_{Random}}) \end{cases}$$

$$\mathrm{RoCDE}_{(\mathrm{Positioin/Speed})} = \begin{cases} \dfrac{\mathrm{AUC_{Spatial}} - \mathrm{AUC_{Random}}}{\mathrm{AUC_{Speed}} - \mathrm{AUC_{Random}}} & (\mathrm{AUC_{Spatial}} \geqq \mathrm{AUC_{Random}}) \\ \dfrac{\mathrm{AUC_{Spatial}} - \mathrm{AUC_{Random}}}{\mathrm{AUC_{RevSpeed}} - \mathrm{AUC_{Random}}} & (\mathrm{AUC_{Spatial}} < \mathrm{AUC_{Random}}) \end{cases}$$

$$\mathrm{RoCDE}_{(\mathrm{Direction/Speed})} = \begin{cases} \dfrac{\mathrm{AUC_{Direction}} - \mathrm{AUC_{Random}}}{\mathrm{AUC_{Speed}} - \mathrm{AUC_{Random}}} & (\mathrm{AUC_{Direction}} \geqq \mathrm{AUC_{Random}}) \\ \dfrac{\mathrm{AUC_{Direction}} - \mathrm{AUC_{Random}}}{\mathrm{AUC_{RevSpeed}} - \mathrm{AUC_{Random}}} & (\mathrm{AUC_{Direction}} < \mathrm{AUC_{Random}}) \end{cases}$$

$AUC_{Spatial}$, AUC_{Speed}, $AUC_{Direction}$, and AUC_{Random} designate the area under the curve (AUC) of the deletion in spatial, speed, direction, and random information order, respectively. $AUC_{RevSpatial}$ is that in the reverse order of spatial information order. The degree of difference of the distribution between an information type (e.g., speed information or direction information) and position information is evaluated by how the deletion order (e.g., blue or

green line) is close to that of random order deletion (black line, independency index = 1) or deletion in the order of spatial information (solid or dotted red line, independency index = 0). If the distribution of an information type is positively or negatively correlated with that of position information (similar to (i) and (ii) in *SI Appendix*, Fig. S8b), the RoCDE would be close to 0. If the distribution patterns of these types of information are independent of each other (similar to (iii) in *SI Appendix*, Fig. S8b), the independency index would be close to 1.

References

1. Leutgeb, J. K., Leutgeb, S., Moser, M.-B. & Moser, E. I. Pattern Separation in the Dentate Gyrus and CA3 of the Hippocampus. *Science* **315**, 961–966 (2007).

2. Shuman, T. *et al.* Breakdown of spatial coding and interneuron synchronization in epileptic mice. *Nature Neuroscience* (2020) doi:10.1038/s41593-019-0559-0.

3. Kropff, E., Carmichael, J. E., Moser, M.-B. & Moser, E. I. Speed cells in the medial entorhinal cortex. *Nature* **523**, 419–424 (2015).

4. Iwase, M., Kitanishi, T. & Mizuseki, K. Cell type, sub-region, and layer-specific speed representation in the hippocampal–entorhinal circuit. *Sci Rep* **10**, 1–23 (2020).

5. Yamasaki, N. *et al.* Alpha-CaMKII deficiency causes immature dentate gyrus, a novel candidate endophenotype of psychiatric disorders. *Mol Brain* **1**, 6 (2008).

6. Butler, L. S. *et al.* Limbic epilepsy in transgenic mice carrying a Ca2+/calmodulin-dependent kinase II alpha-subunit mutation., Limbic epilepsy in transgenic mice carrying a Ca2+/calmodulin-dependent kinase II alpha-subunit mutation. *Proc Natl Acad Sci U S A* **92, 92**, 6852, 6852–6855 (1995).

7. Shin, R. *et al.* The immature dentate gyrus represents a shared phenotype of mouse models of epilepsy and psychiatric disease. *Bipolar Disord* 405–21 (2013) doi:10.1111/bdi.12064.

8. Cho, Y. H., Giese, K. P., Tanila, H., Silva, A. J. & Eichenbaum, H. Abnormal Hippocampal Spatial Representations in αCaMKIIT286A and CREBαΔ− Mice. *Science* **279**, 867–869 (1998).

9. Rotenberg, A., Mayford, M., Hawkins, R. D., Kandel, E. R. & Muller, R. U. Mice Expressing Activated CaMKII Lack Low Frequency LTP and Do Not Form Stable Place Cells in the CA1 Region of the Hippocampus. *Cell* **87**, 1351–1361 (1996).

10. Antoine, M. W., Langberg, T., Schnepel, P. & Feldman, D. E. Increased Excitation-Inhibition Ratio Stabilizes Synapse and Circuit Excitability in Four Autism Mouse Models. *Neuron* **0**, (2019).

11. Gonçalves, J. T., Anstey, J. E., Golshani, P. & Portera-Cailliau, C. Circuit level defects in the developing neocortex of Fragile X mice. *Nat Neurosci* **16**, 903–909 (2013).

12. Goel, A. *et al.* Impaired perceptual learning in a mouse model of Fragile X syndrome is mediated by parvalbumin neuron dysfunction and is reversible. *Nat Neurosci* **21**, 1404–1411 (2018).

13. Wood, E. R., Dudchenko, P. A., Robitsek, R. J. & Eichenbaum, H. Hippocampal Neurons Encode Information about Different Types of Memory Episodes Occurring in the Same Location. *Neuron* **27**, 623–633 (2000).

14. Kay, K. *et al.* Constant Sub-second Cycling between Representations of Possible Futures in the Hippocampus. *Cell* (2020) doi:10.1016/j.cell.2020.01.014.

15. Dragoi, G. & Tonegawa, S. Distinct preplay of multiple novel spatial experiences in the rat. *Proc Natl Acad Sci U S A* **110**, 9100–9105 (2013).

16. Yamamoto, J., Suh, J., Takeuchi, D. & Tonegawa, S. Successful Execution of Working Memory Linked to Synchronized High-Frequency Gamma Oscillations. *Cell* **157**, 845–857 (2014).

17. Buzsaki, G., Horvath, Z., Urioste, R., Hetke, J. & Wise, K. High-frequency network oscillation in the hippocampus. *Science* **256**, 1025–1027 (1992).

18. Buzsáki, G. Hippocampal sharp wave-ripple: A cognitive biomarker for episodic memory and planning. *Hippocampus* **25**, 1073–1188 (2015).

19. Ziv, Y. *et al.* Long-term dynamics of CA1 hippocampal place codes. *Nature Neuroscience* **16**, 264 (2013).

20. Shoji, H., Hagihara, H., Takao, K., Hattori, S. & Miyakawa, T. T-maze Forced Alternation and Left-right Discrimination Tasks for Assessing Working and Reference Memory in Mice. *J Vis Exp* (2012) doi:10.3791/3300.

21. Skaggs, W. E., McNaughton, B. L. & Gothard, K. M. An Information-Theoretic Approach to Deciphering the Hippocampal Code. in *Advances in Neural Information Processing Systems 5* (eds. Hanson, S. J., Cowan, J. D. & Giles, C. L.) 1030–1037 (Morgan-Kaufmann, 1993).

22. Stefanini, F. *et al.* A Distributed Neural Code in the Dentate Gyrus and in CA1. *Neuron* (2020) doi:10.1016/j.neuron.2020.05.022.

23. Glaser, J. I. *et al.* Machine Learning for Neural Decoding. *eNeuro* **7**, (2020).

24. Ravassard, P. *et al.* Multisensory Control of Hippocampal Spatiotemporal Selectivity. *Science* **340**, 1342–1346 (2013).
